# Checklist and Review of Population Genetic Studies with Molecular Markers Applied to the Wild Cat Species Present in Colombia for Conservation Purposes

**DOI:** 10.3390/ani16040629

**Published:** 2026-02-16

**Authors:** Manuel Ruiz-García

**Affiliations:** Laboratorio de Genética de Poblaciones Molecular-Biología Evolutiva, Unidad de Genética, Departamento de Biología, Facultad de Ciencias, Pontificia Universidad Javeriana, Bogota 110311, Colombia; mruizgar@yahoo.es or mruiz@javeriana.edu.co

**Keywords:** biological conservation, jaguar, jaguarundi, margay, neotropics, ocelot, Pampas cat, phylogeography, population genetics, puma, tigrina

## Abstract

At least seven species of wild cats inhabit Colombia, and these species are also distributed throughout Mexico, Central America, and the rest of South America (jaguar, puma, jaguarundi, ocelot, margay, tigrina, and Pampas cat). A checklist and review of phylogeographic and population genetic studies on these seven wild cat species has been conducted here, as this information is vital for effective conservation programs. The jaguar is the feline species on which the most population genetic studies have been carried out in the Neotropics. In general, little genetic structure has been found at a macro-geographic scale. Genetic diversity is high in countries such as Colombia, Peru, and Bolivia, and generally throughout the Amazon basin. However, genetic diversity is more moderate or even significantly lower in Mexico and the Brazilian Atlantic Forest. Much of the genetic research on the jaguar has focused on Brazil, Mexico, and Belize, but Colombia is also very well represented in these studies. However, there is a complete or very pronounced lack of data in other areas such as Venezuela, the Guianas, some Central American countries, Paraguay, and northern Argentina. After the jaguar, the most studied feline in Neotropics from a population genetics perspective is the puma. In North America, this species has low genetic diversity, while the diversity in Central America is moderate, and South America is where genetic diversity is highest. The countries best represented in these studies are Brazil (southern of the country), Mexico, Belize, and Argentina. However, countries like Colombia, Ecuador, Peru, and Paraguay are very poorly represented in puma genetic studies. Very few genetic studies have been conducted on the jaguarundi, despite its vast geographic distribution. In northern Mexico, its genetic diversity is very low, but in countries like Colombia, Peru, and Bolivia, genetic diversity is very high. Colombia is probably the country where jaguarundis have been genetically studied most extensively. The third wild cat species with the most molecular studies in Neotropics is the ocelot, although it lags considerably behind jaguars and pumas. Its genetic diversity is low in Texas and northern Mexico, but very high, especially in countries surrounding the Amazon basin. A good number of macro-geographic studies have been conducted on the ocelot, and these studies are very representative of ocelots in countries such as Colombia (probably the best represented country), Ecuador, Peru, Bolivia, Panama, and Costa Rica. However, there are other countries where molecular studies of the ocelot have not been carried out, such as Paraguay and Argentina, with the lack of such studies in Brazil being particularly noteworthy. Very few molecular studies have been conducted on the margay. In general, its genetic diversity is very high in all the studies performed. Colombia, Peru and Bolivia are very well represented, but the lack of molecular studies in Mexico, much of Central America, and South American countries such as Brazil, Paraguay, and Argentina is striking. The tigrina is one of the Neotropical wild cat species that requires the most molecular studies to unravel its complex systematics. Only the southern Brazilian tigrina (*Leopardus guttulus*), which was elevated to a new species, has generated comprehensive molecular information. Molecular studies of the Andean tigrina have revealed a very complex picture that, at present, does not allow us to know exactly how many species or taxa inhabit that area of South America and, therefore, to develop a conservation program that adequately correlates with that number of taxa. Finally, in the case of the Pampas cat, molecular studies are well represented with specimens from Peru, Bolivia, Chile, Argentina, Brazil and Uruguay. Studies are needed in Paraguay, and especially in Ecuador and southern Colombia (assuming a stable population of Pampas cats exists in the latter country), where even at the molecular level, the specific taxon (one species or five species) present has not yet been determined. All this information is essential for developing effective regional and global conservation programs for these magnificent creatures. In Colombia, the development of molecular studies with the puma, the tigrina, and the Pampas cat is of special interest.

## Simple Summary

Felines are of vital importance from a biological conservation perspective because they play a crucial role in the food chains of the ecosystems they inhabit, in addition to being iconic organisms greatly admired by humans. Colombia, one of the world’s most biodiverse countries, is home to at least seven species of wild cats. For this country, the biological conservation of these felines is a vital objective. One of the most important aspects for the conservation of these species is having a comprehensive understanding of their population genetics, as well as a clear systematic classification of these feline species. Here we review all the phylogeographic and population genetic studies that have been carried out with these seven species of felines (jaguar, puma, jaguarundi, ocelot, margay, tigrina, and Pampas cat) in Latin America (including Colombia) to determine which geographic areas and population genetic aspects are already known about these felines and which are still to be determined and which are essential for, both in Colombia and in Latin America, effective conservation programs for these admired and fascinating creatures that are wild cats.

## 1. Introduction

Since immemorial times, wild cats have held a powerful attraction for humans due to their beauty, behavior, and formidable predatory nature. Furthermore, they are frequently of paramount importance in food chains and, therefore, are emblematic and iconic species in many aspects of biological conservation [1,2,3]. A crucial aspect for the effective biological conservation of these beautiful and important carnivores lies in the molecular genetic understanding of the fundamental factors that modulate their population genetics, phylogeography, and phylogenetic relationships. Among the genetic factors relevant to feline conservation are: (1) estimating the levels of genetic diversity in species and populations; (2) the determination of geographically differentiated gene pools (phylogeography), which may give rise to management units (MUs) or evolutionarily significant units (ESUs) [4]; (3) the degree of genetic heterogeneity and/or gene flow among their populations (connectivity), in short, their genetic and spatial structure; (4) the interspecific phylogenetic relationships with the corresponding estimates of their divergence times; (5) their current and historical effective numbers, which are important components of Population Viability Analyses; (6) the demographic changes that have occurred in the environments they inhabit, whether historical (correlated with climatological, geological and hydrological changes basically during the Pliocene and Pleistocene) or current (recent anthropogenic pressure due to habitat fragmentation or direct hunting); and (7) the geographical assignment of hunted, seized or captive specimens, as well as their correct taxonomic affiliation.

**Figure 1:**
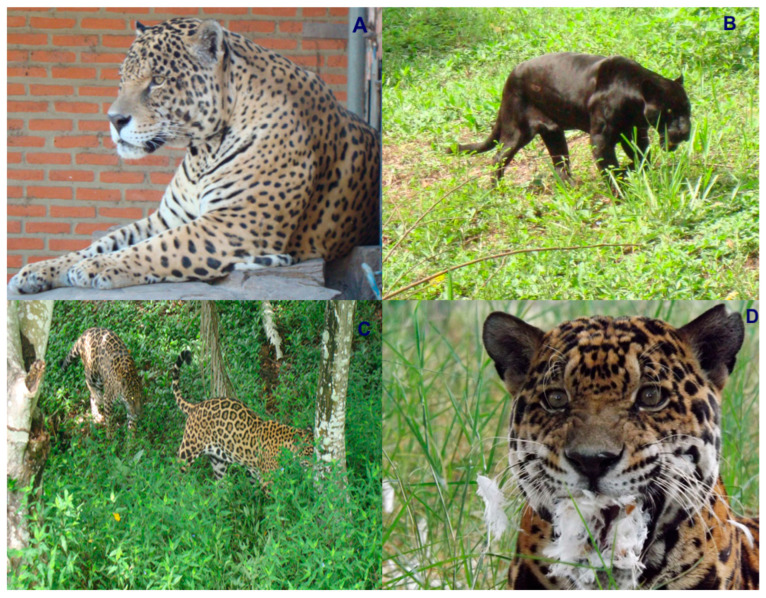
Jaguar (*Panthera onca*). (**A**) A jaguar in captivity in Santa Cruz de la Sierra (Bolivia). (**B**) A melanic jaguar near to Pucallpa (Ucayali River) (Peru). (**C**) Two jaguars near to Pucallpa (Ucayali River) (Peru). (**D**) A jaguar devouring prey near to Guayaquil (Ecuador).

**Figure 2:**
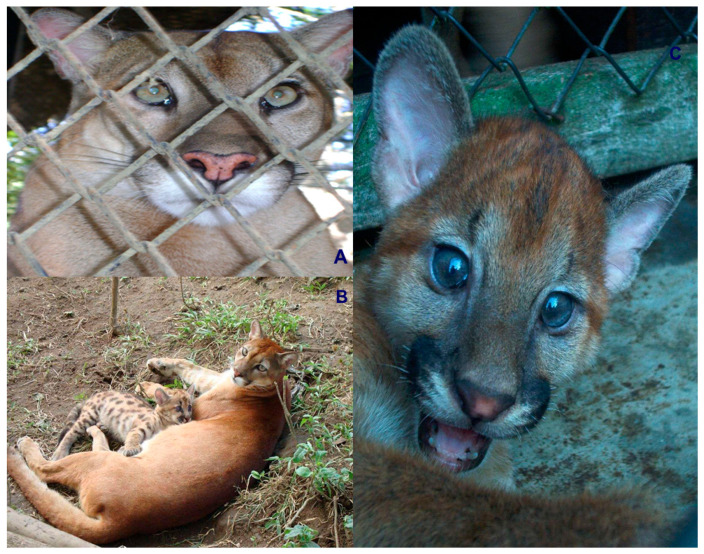
Puma (*Puma concolor*). (**A**). A captive puma in Pucallpa Zoo (Peru). (**B**) A mother puma with her cub, near Macas (Ecuador). (**C**) A baby puma in the Quistococha Zoo near to Iquitos (Peru).

**Figure 3:**
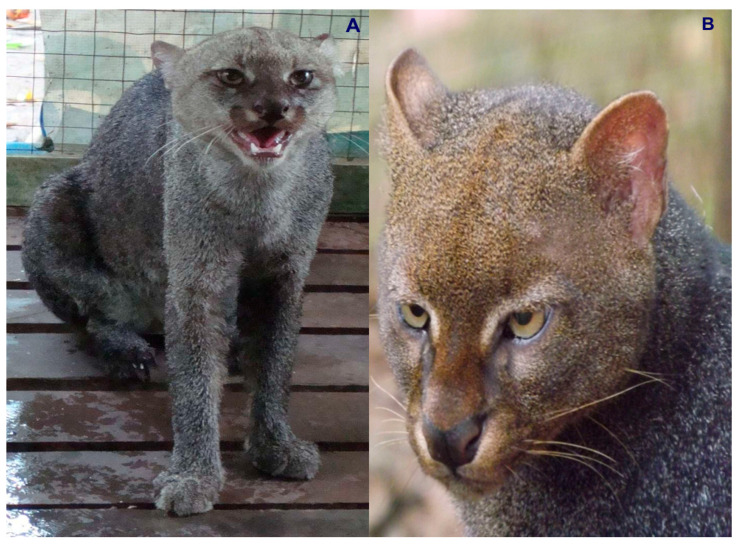
Jaguarundi (*Puma = (Herpailurus) yagouaroundi*). (**A**) A jaguarundi in Yarinacocha (Ucayali River) (Peru). (**B**) A jaguarundi in the Napo province (Ecuador).

**Figure 4:**
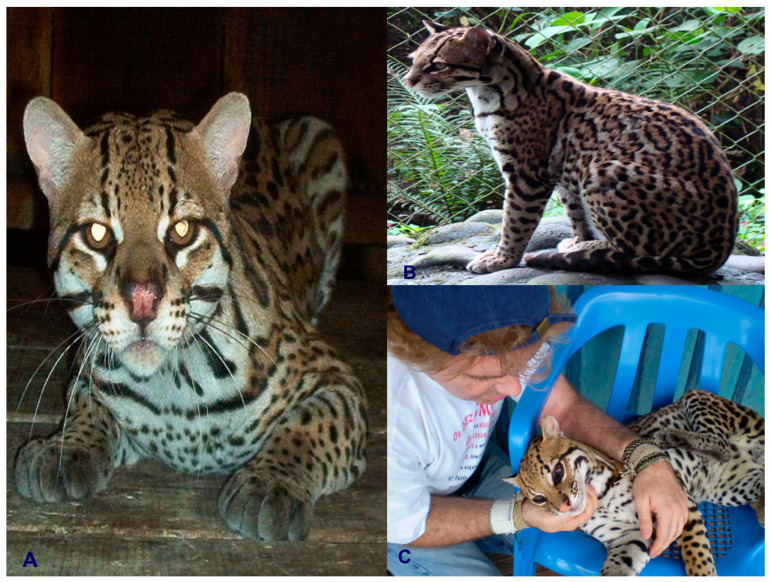
Ocelot (*Leopardus pardalis*). (**A**) An ocelot male in captivity in Quistococha Zoo near to Iquitos (Peru). (**B**) An ocelot in captivity near to Macas (Ecuador). (**C**) The author with an ocelot in Yahuma (Amazon River; Loreto department; Peru).

**Figure 5:**
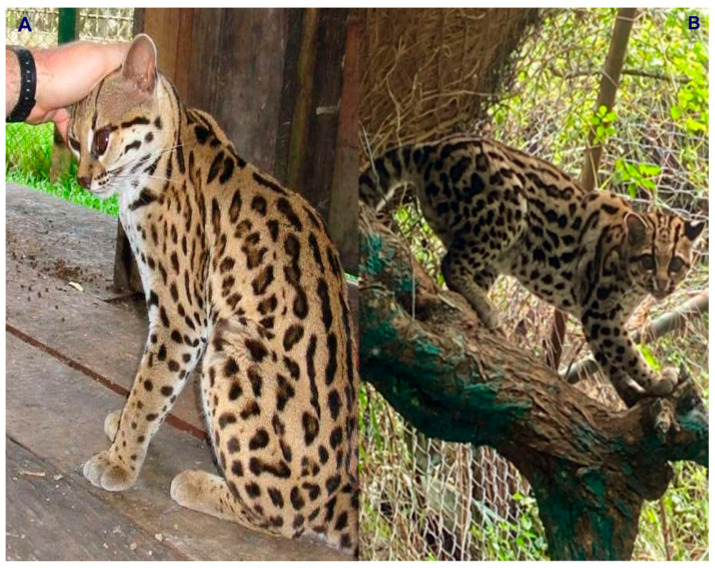
Margay (*Leopardus wiedii*). (**A**) A margay in captivity in Leticia (Amazonas department, Colombia). (**B**) A margay in captivity in the Totorilla Zoo (Ayacucho, Peru).

**Figure 6:**
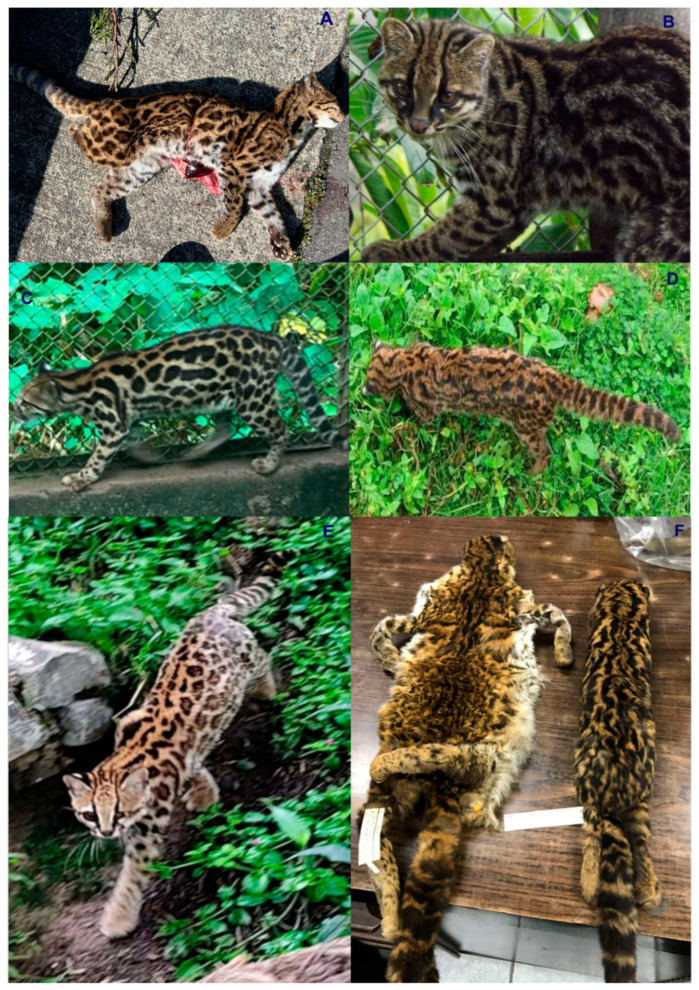
Tigrina (*Leopardus tigrinus*). All the images are from northern Andean tigrina. Different morphotypes were observed. (**A**) A tigrina runs over in Caldas department (Colombia). (**B**) A tigrina captured in Imbabura (Ecuador). (**C**) A tigrina captured in Tingo María (Huánuco department, Peru). (**D**) A tigrina in Cotopaxi province (Ecuador). (**E**) A tigrina in the Totorilla Zoo (Ayacucho, Peru). (**F**) Two different skins of tigrinas from northern Ecuador. The differences in fur between these specimens are clearly observable.

**Figure 7:**
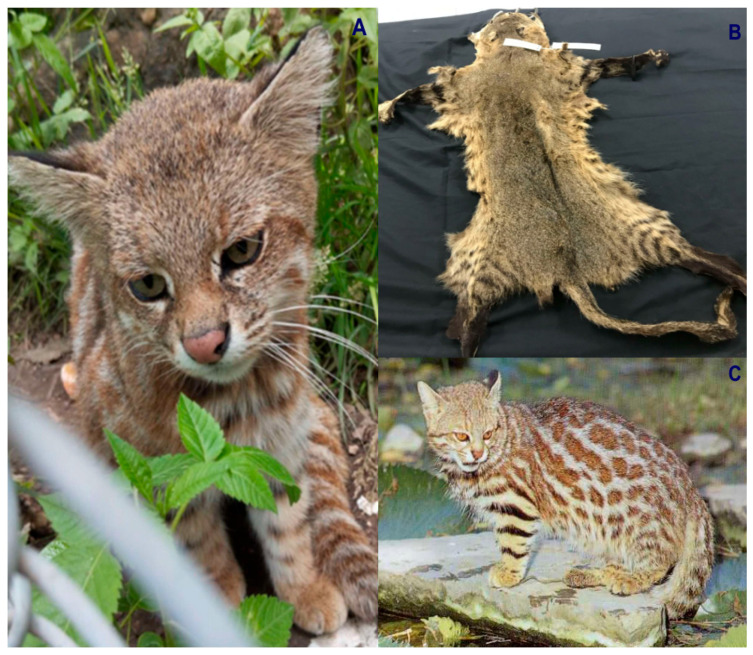
Pampas cat (*Leopardus colocola*). (**A**) A Pampas cat (*garleppi* form) from the Totorilla Zoo (Ayacucho, Peru). (**B**) A skin of a Pampas cat in the Noel Kempff Museum in Santa Cruz de la Sierra (Bolivia) (*braccatus* form). (**C**) A Pampas cat in the Ica Zoo (Peru) (*garleppi* form).

Colombia is ranked as the fourth most biodiverse country on the planet (at least 80,000 registered species, although there could be between 200,000 and 900,000 species [5]), after Brazil, Indonesia, and China. It also ranks first in the number of orchids, lepidopteran, and bird species; second in the number of palms, freshwater fish, amphibian, and bat species; and third in the number of overall plant species. Additionally, Colombia ranks sixth globally in the number of mammal species [6]. In this country, at least seven species of wild cats have been recorded, representing an important component of the Neotropical mammal fauna of this South American nation. These species include the jaguar (*Panthera onca*) (Figure 1), the puma (*Puma concolor*) (Figure 2), the jaguarundi (*Puma* = (*Herpailurus*) *yagouaroundi*) (Figure 3), and the small or medium-sized spotted cats of the ocelot lineage: the ocelot (*Leopardus pardalis*) (Figure 4), the margay (*Leopardus wiedii*) (Figure 5), the tigrillo or oncilla (*Leopardus tigrinus*, or *Leopardus pardinoides*) (Figure 6), and the pampas cat (*Leopardus colocola*, *or Leopardus garleppi*) (Figure 7). In the case of the last two taxa, as we will discuss in detail later, the taxonomic nomenclature, or even the number of taxa, is not entirely clear.

The comparison between the values of genetic diversity [here measured as expected heterozygosity (H_e_), the average number of alleles per locus (n_A_), or polymorphism information content (PIC), in the case of nuclear markers, or haplotype diversity (H_d_), and nucleotide diversity, π, in the case of mitochondrial markers], degree of genetic structure, number of genetically differentiable pools, existence of significant spatial structure, effective numbers, divergence times between populations, and possible demographic changes for these seven wild cat taxa in Colombia with respect to these same variables and species found in other regions of the Neotropics, is of great help to the conservation programs of these cats in the Colombian territory. It is interesting to note that H_e_ and H_d_ values range from 0 to 1. Values above 0.7 indicate high genetic diversity, and values close to or higher than 0.9 indicate very high genetic diversity. Furthermore, π values above 1 or 2% are considered indicative of high nucleotide diversity. On the other hand, the most widely used statistic for estimating genetic heterogeneity among populations or taxa is Wright’s fixation index (F_ST_). This statistic ranges from 0 to 1. Values between 0 and 0.05 indicate little genetic differentiation; values between 0.05 and 0.15 are consistent with moderate genetic differentiation; values between 0.15 and 0.25 denote important genetic differentiation; and values above 0.25 indicate very high genetic differentiation [7,8].

Therefore, this review presents a comprehensive overview of molecular population genetic studies conducted in Latin America, and specifically in Colombia, on the various feline species inhabiting this country, as previously mentioned. Many of these studies have helped to estimate or determine some of the basic parameters that shape the genetic structure of these feline species. The review will focus on the seven taxa mentioned, following the three genetically distinct feline lineages [9,10] present in the Neotropics and Colombia (*Panthera*, *Puma*, and *Leopardus*), and within each lineage, beginning with the largest species and ending with the smallest.

## 2. Results of the Most Important Molecular Population Genetic Studies on the Seven Wild Cat Taxa Existing in Colombia

### 2.1. Jaguar

The jaguar (*Panthera onca*) is the only species of the genus *Panthera* present in the Americas and is the largest wild cat in this continent (weight between 45 and 160 kg and body length, excluding the tail, between 1.12 and 1.85 m). Globally, *P. onca* is listed as Near Threatened under criteria A2cd; the current population trend is decreasing, and the population is severely fragmented.

Several subspecific jaguar classifications have been proposed [11,12,13]. The current subspecific classification is that employed by Seymour [14], and it is mainly based on [13]. The subspecies described are as follows:

(1) *Panthera onca arizonensis* (Goldman, 1932) could be distributed from eastern Arizona north to the Grand Canyon and south to New Mexico (USA) and northeastern Sonora (Mexico). It was described as the largest of the northern jaguar subspecies.

(2) *Panthera onca hernandesii* (Gray, 1857) could be distributed in western Mexico from Sinaloa to Tehuantepec and from Sinaloa to Louisiana. Nelson and Goldman [12] and Pocock [13] stated that the skulls of this subspecies are smaller than that of *P. o. arizonensis* but like *P. o. centralis* and larger than *P. o. goldmani*.

(3) *Panthera onca veraecrucis* (Nelson and Goldman, 1933) could be distributed in eastern Mexico from Tabasco to Veracruz and from Tamaulipas to central Texas.

(4) *Panthera onca goldmani* (Mearns, 1901) could be found in Belize, northern Guatemala until Campeche and Yucatan in Mexico. The studies of Mearns [11], Nelson and Goldman [12] and Pocock [13] showed that the skulls of this putative subspecies have similar shape to *P. o. centralis*, but smaller.

(5) *Panthera onca centralis* (Mearns, 1901) could be found in northern, central and western Pacific coast of Colombia and through Panama, Costa Rica, Nicaragua, El Salvador and Honduras. Nelson and Goldman [12] described this subspecies as like *P. o. hernandesii* but with less depressed nasals.

(6) *Panthera onca onca* (Linnaeus, 1758) could be found from Venezuela to Guianas in its northern distribution, eastern Brazil, all the Amazon Basin including Colombia, Peru, Ecuador, Bolivia and Brazil, from Pernanbuco and Rio Grande do Sul in southern Brazil and from northern Matto Grosso, Brazil to the Santa Cruz department in Bolivia. This is the largest subspecies along together with *P. o. paraguensis*, but with a slightly smaller skull, elevated less frontal region and heavy dentition [15]. Pocock [13] recognized this subspecies by its distinctive condylobasal lengths.

(7) *Panthera onca peruviana* (DeBlainville, 1843) could be distributed by the coastal areas of Peru and Ecuador. However, the real status of this subspecies is not clear because very few skulls have been studied [12,13].

(8) *Panthera onca paraguensis* (Hollister, 1914) could be found from the southern Matto Grosso, Parana and Sao Paulo States (Parana River Valley) in Brazil to Paraguay and northeastern Argentina. This subspecies was named by Pocock [13] as *P. o. palustris* (Ameghino, 1888), based on an analysis of one fossil rather than on an extant form.

**(1)-** The first molecular study of the jaguar, on a global scale of its geographic distribution in the Neotropics, was that of Eizirik et al. [16], who sequenced 715 base pairs (bp) of the mitochondrial DNA (mtDNA) control region for 37 specimens and, in addition, obtained the genotypes of 29 nuclear microsatellite loci for 42 specimens mainly from zoos in various Latin American countries. None of these specimens were of Colombian origin. This study revealed a very high haplotype diversity (H_d_ = 0.939 ± 0.026) for the entire set of jaguars analyzed, but a relatively low nucleotide diversity (π = 0.0077 ± 0.0001) for the mitochondrial marker used, and high values for nuclear genetic diversity (H_e_ = 0.739 and n_A_ = 8.31). MtDNA detected two weakly supported phylogeographic groups due to the presence of the Amazon River. North of the river, a group comprised Mexican, Central American, and northern South American specimens, and south of the river, a group comprising specimens from Peru, Brazil, Bolivia, and Paraguay. However, some gene flow was detected between these two groups. This weak structure, with low inter-regional differentiation, was inferred to have been caused by a rather recent population expansion, around 300,000 years ago, followed by a history of demographic connectivity over a continental scale. Microsatellite markers detected a slight differentiation between the Mexican and Central American population compared to that of northern South America, presumably due to the Darién Gap. However, this structuring is weak. Some haplotypes from northern South America showed that they could be the origin of all the haplotypes found in this study. The separation between the different mitochondrial lineages of these jaguars was estimated to be between 280,000 and 510,000 years. **(2)-** Moreno et al. [17] analyzed 39 jaguars sampled from Brazilian zoos using four nuclear microsatellites. Three out of four loci showed high levels of allele diversity for the jaguars from these Brazilian zoos, with PIC = 0.59 ± 0.125 and n_A_ = 9 ± 3.67. **(3)-** Another interesting study was carried out by Soares et al. [18] using seven microsatellites for the analysis of four jaguars involved in an infanticide event. Two were dead cubs and two were adult males. The analysis showed that the male who killed the two cubs was assigned as the true sire. **(4)-** Eizirik et al. [19] analyzed samples from 23 jaguars sampled in two locations in the Pantanal (Caiman Ecological Refuge and San Francisco Ranch; Mato Grosso do Sul state, Brazil) for 12 microsatellite loci, this being the first study carried out with the jaguar at a microgeographic scale. The genetic diversity found ranged from moderate to high (depending on each marker) with an average H_e_ value of 0.717 and an n_A_ value of 5.83, which can be considered high. Additionally, these authors estimated the probability of identity (PID) using these 12 microsatellite loci for the overall Pantanal sample, which was 2 × 10^−13^, indicating that it is extremely unlikely that any two jaguar specimens may bear the same composite genotypes at these 12 microsatellite loci (as will be discussed later, this aspect had already been shown by Ruiz-García et al. [20]). **(5)-** Haag et al. [21] studied melanism in jaguars by directly genotyping the molecular polymorphism underlying this coloration trait, since a 15 bp deletion in the jaguar *MC1R* gene (the “*MC1R-D15*” allele) was perfectly associated with melanism [22]. For this purpose, the excrement of two jaguars collected in Ivinhema State Park (Mato Grosso do Sul; Brazil), two others in Iguazu National Park (Paraná; Brazil) and five in the Misiones province (Argentina) was analyzed, all of which were homozygous for the wild-type allele of 173 bp. Another 14 jaguars from Cantao State Park (Tocantins, Amazon, Brazil) were also homozygous for the wild-type allele of 173 bp, but one individual from this same population was found to be a 158/173 bp heterozygote and, therefore, had melanistic fur. Other relevant studies carried out with this species outside of Colombia were the following: **(6)-** Haag et al. [23] analyzed four remnant jaguar populations in the Brazilian Atlantic Forest using 13 nuclear microsatellites and 50 samples, finding significant genetic heterogeneity among these populations (F_ST_ = 0.048–0.198) with evidence of recent allele loss in some of them. The effective population size (N_e_) ranged from 4.6 to 51.4 animals, showing a significant effect of genetic drift due to the extreme reduction in habitat in that area of Brazil. **(7)**- Roques et al. [24] systematically used fecal analysis for DNA extraction for the first time. They identified 30 jaguars from the Brazilian Caatinga (n = 16) and Pantanal (n = 14) and analyzed them for 11 microsatellite loci. For the first population, they estimated H_e_ = 0.70, and for the second population, H_e_ = 0.67. The genetic differentiation between the two populations was significant (F_ST_ = 0.178, *p* < 0.01). **(8)**- Valdez et al. [25] analyzed 52 jaguars from four locations in the southern Pantanal of Brazil using 12 nuclear microsatellites. Evidence of some internal structure was found (F_ST_ = 0.045, *p* = 0.001), but, in general, the genetic diversity found was high (Caiman Ecological Refuge, n = 22, H_e_ = 0.66 ± 0.17, n_A_ = 5.67 ± 1.69; San Francisco Ranch, n = 11, H_e_ = 0.71 ± 0.12, n_A_ = 4.5 ± 1.5; San Bento Ranch, n = 10, H_e_ = 0.70 ± 0.18, n_A_ = 5.0 ± 1.68; Sete Ranch, n = 10, H_e_ = 0.69 ± 0.14, n_A_ = 4.75 ± 1.48; global value for the Pantanal, n = 52, H_e_ = 0.70 ± 0.15, n_A_ = 6.55 ± 2.64) and the analyzed area comprised a single verifiable gene pool, unlike that recorded in the Brazilian Atlantic Forest using the STRUCTURE program [26]. **(9)**- Wultsch et al. [27] examined the levels of gene flow and connectivity among 115 jaguars located in Mesoamerica (Mexico, Belize, Guatemala, Honduras, and Costa Rica) for 12 nuclear microsatellites. The level of genetic diversity was moderate (H_e_ = 0.59 ± 0.04, n_A_ = 4.50 ± 1.05), with the Mexican sample showing the lowest genetic diversity (H_e_ = 0.54, n_A_ = 3.25) and the Costa Rican sample showing the highest level (H_e_ = 0.64, n_A_ = 6.0). Genetic heterogeneity was significant among these populations (F_ST_ = 0.09–0.15), and various principal component and Bayesian assignment analyses distinguished four genetically distinct clusters with varying levels of admixture among them. **(10)**- Wultsch et al. [28] analyzed data from 14 polymorphic microsatellite loci for 1053 scat samples collected from wild jaguars, pumas, and ocelots in Belize. They assessed levels of genetic diversity, potential genetic clusters, and gene flow estimates for the three cat species. The jaguars (n = 65) showed the lowest genetic diversity estimates (H_e_ = 0.57 ± 0.02 and n_A_ = 3.80 ± 0.34), followed by pumas (n = 54) (H_e_ = 0.57 ± 0.08 and n_A_ = 4.46 ± 1.28), and ocelots (n = 30) (H_e_ = 0.63 ± 0.03 and n_A_ = 5.11 ± 0.15). They observed low to moderate levels of genetic differentiation for all three target cat species, with jaguars showing the lowest degree of genetic subdivision across the country. **(11)**- Rueda-Zozaya et al. [29] emphasized the importance of maintaining an adequate level of genetic variation and proposed the implementation of standardized studbooks for jaguars in Mexico, mainly to avoid inbreeding. They analyzed 56 jaguars from 14 Mexican zoos for 11 microsatellite loci. The genetic diversity was moderate (H_e_ = 0.65 ± 0.08 and n_A_ = 5.03), and using the STRUCTURE program, three different gene pools were detected for these captive Mexican jaguars. The overall N_e_ ranged from 13.4 to 22.7 individuals, and significant bottlenecks were detected both in the total captive population and in the three genetically distinct groups.

**(12)**- Roques et al. [30] compared the genetic structure of Mexican and Brazilian jaguar populations with 11 microsatellite loci. To do this, they obtained DNA, basically, from fecal samples in four localities of the Brazilian Amazon (12 different specimens), one locality of the Brazilian caatinga (18 different specimens), three localities of the Brazilian Cerrado (14 different specimens) and one locality of the Brazilian Pantanal (34 different specimens) for a total of 78 different jaguars, while for Mexico they studied six different localities with a total of 24 different jaguars (total sample size: 102 jaguars). Genetic diversity was significantly higher for Brazilian jaguars (H_e_ = 0.812 ± 0.053; n_A_ = 9.45 ± 0.829) than for Mexican jaguars (H_e_ = 0.654 ± 0.147; n_A_ = 4.45 ± 0.325). Analysis using STRUCTURE for Brazil detected four distinct populations (Amazon, Pantanal, Caatinga, and a transition zone in central Brazil). For Mexico and Belize, only one population, highly differentiated from the Brazilian populations, was detected. STRUCTURE identified 17 migratory jaguars in Brazil: 14 in the central region, two in the Pantanal, and one in the Amazon. The GENECLASS program [31] identified nine migratory jaguars in Brazil, with both programs detecting four jaguars from central Brazil as first-generation migrants. Only the jaguar populations of the Caatinga and Mexico showed any evidence of a bottleneck in the infinite allele mutational (IAM) model (for the stepwise mutational model, SMM, no jaguar population showed evidence of a bottleneck), and only the Caatinga population did so in the M-ratio analysis [32]. The application of the 2Mod program [33] determined that the jaguar population of the Brazilian Caatinga correlates with a pure genetic drift model and that the total isolation of this population has occurred in the last 20 years. Effective numbers were estimated using the LDNE program [34] and the ONESAMP program [35], both showing that the Amazonian population is the one that could possess the highest N_e_ values (21–∞ and 298, respectively), while the values for the Mexican sample (14–45 and 22–38, respectively), the Pantanal sample (10–28 and 10–17, respectively), and the Caatinga sample (7–28 and 12–16, respectively) were considerably lower.

**(13)**- Zanin et al. [36] analyzed the influence of distance isolation in Mexican jaguars and pumas. From DNA extracted from feces, 11 microsatellite loci were analyzed in jaguars (n = 34) and 12 microsatellite loci in pumas (n = 66). Some isolation-by-distance was detected for both species in Mexico, but the patterns of isolation-by-distance were more pronounced for the puma than for the jaguar using sPCA (spatial principal component analysis). STRUCTURE detected two populations for pumas and eight for jaguars according to Δk, while it detected five puma populations and an undetermined number for jaguars using LnP(D). The TESS program [37] detected two populations for both species. Genetic diversity for pumas and jaguars was similar (pumas: H_e_ = 0.655 ± 0.074 and n_A_ = 3.045 ± 0.850; jaguars: H_e_ = 0.610 ± 0.036 and n_A_ = 2.870 ± 0.279), with these values being moderate. **(14)**- Srbek-Araujo et al. [38] used noninvasive fecal sampling and microsatellite markers to investigate the genetic diversity of jaguars in one of the last remaining populations of this species in the entire Brazilian coastal on the Atlantic Forest. They analyzed 11 different jaguars for 11 microsatellite loci sampled at the Reserva Natural Vale (Espíritu Santo state, Brazil). They observed low levels of genetic diversity (H_e_ = 0.532 ± 0.203 and n_A_ = 3.45 ± 1.23) and they estimated a very small effective size for this population (N_e_ = 7.9 individuals). They performed comparative analyzes incorporating data from previously surveyed populations located farther inland in the Atlantic Forest in Brazil, revealing that the Brazilian coastal population studied showed significant genetic differentiation by using the STRUCTURE program, with F_ST_ values ranging from 0.279 to 0.133 (*p* < 0.001). Despite its low genetic diversity (due to extreme habitat destruction in this area of Brazil and the resulting significant gene drift), the authors detected potentially unique alleles in the coastal Brazilian jaguar population, highlighting its importance in maintaining the remaining genetic variability of jaguars in the Brazilian Atlantic Forest. **(15)**- Menchaca et al. [39] analyzed 50 jaguars for 12 nuclear microsatellites in Belize. The level of genetic diversity was moderate (H_e_ = 0.603 ± 0.207, and n_A_ = 5 ± 2.16), and a single jaguar gene pool was identified in this Central American country using the STRUCTURE, TESS, and Geneland [40] programs. **(16)**- Lorenzana et al. [41] analyzed 73 jaguars from various regions of the Brazilian Amazon using 11 nuclear microsatellites and compared their genetic structure with that of jaguars previously studied in the Atlantic Forest and the Brazilian Pantanal. Genetic diversity in the Brazilian Amazon was high (H_e_ = 0.768 ± 0.134, and n_A_ = 11.0 ± 5.67), and consistently higher than that found in the other two areas of Brazil. No genetic subdivisions were detected in the Brazilian Amazon (F_ST_ = 0.037–0.052, not significant), indicating long-scale connectivity over distances greater than 3000 km. No significant spatial autocorrelation was found. Three programs were used for the estimation of effective numbers (LDNE, NeESTIMATOR v2.1, [42], and SPEED-NE v2.3, [43]). The 95% parametric confidence intervals showed for the Amazon jaguar population values of 241.4–∞, with a point estimate of 724 (NeESTIMATOR), of 257.4–∞, with a point estimate of 887.7 (LDNE), and of 116.3–∞, with a point estimate of 278 (SPEED-NE), these values being substantially higher than those found in the Pantanal, 79.8 (53.3–141.4), 81.2 (54.0–145.6), and 50.5 (43.8–59.6), respectively, and extremely higher than those found in the Brazilian Atlantic Forest, 20.4 (17.8–23.5), 20.5 (17.8–23.6), and 16.9 (14.3–20.73), respectively. **(17)**- Zanin et al. [44] studied whether the current genetic structure of the two large American felids, jaguar and puma, was mediated by changes in climatic suitability and connection routes across modern and paleoclimatic landscapes. To do this, they obtained DNA from scat samples collected from both species at 15 locations (jaguar) and 20 locations (puma), where they were able to differentiate 71 jaguars and 106 pumas using 11 and 12 microsatellite loci, respectively. The samples came from Mexico and Belize, Brazil, and, incidentally, some from the Bolivian Chaco. They estimated species distribution under five climatic landscapes (modern, Holocene, last Pleistocene maximum glaciations, average suitability, and climatic instability) and correlated them with genetic isolation through causal modeling on a resemblance matrix. Both species exhibit genetic isolation patterns correlated with Pleistocene glacial maximum climatic suitability, suggesting that these areas may have functioned as “allele refuges.” However, the jaguar showed greater vulnerability to climate change, responding to modern climatic suitability and connecting routes, whereas the puma showed a continuous and gradual transition of genetic variation. Despite differential responsiveness to climate change, both species are subject to climatic effects on genetic configuration, which may make them susceptible to future climatic changes. **(18)**- Lorenzana et al. [45] analyzed the complete genomes of 13 different jaguars, 11 from Brazil and two from Mexico. Clearly, the Brazilian animals (Amazon and Pantanal) presented a high SNV rate of heterozygotes (0.12), while the two Mexican specimens presented considerably lower levels of genetic diversity in their complete genomes (0.06–0.08), as did two specimens from the Brazilian Atlantic Forest. This study also detected a population decline around 500,000 years ago. Subsequently, a gradual population increase was observed until 30,000 years ago, when another population decline began, more pronounced in the Mexican population.

In Colombia, several molecular studies have been conducted on the jaguar: **(1)**- the first molecular analysis was carried out by Ruiz-García [46], who estimated the genetic diversity of 24 jaguars of Colombian origin (some specimens from the northern part of the country, departments of Córdoba and Bolívar, although most came from the department of Amazonas) using five microsatellites, showing a value of H_e_ = 0.76 ± 0.29, very similar to that reported in the same year by Eizirik et al. [16]. The n_A_ value was 5 ± 1.4. This study did not detect any bottleneck events for Colombian jaguars regardless of the mutational model used for microsatellites. Based on various demographic models, it was estimated that, for the jaguar, the N_e_/N ratio (N_e_ = effective number; N = total number of individuals in a population or species) would range between 0.5 and 0.67. Using two different mutational models (IAM and SMM, which represent the most extreme mutational models), historical N_e_ values for the jaguar in the Amazon were obtained (including, in addition to Colombian specimens, others sampled in Ecuador, Peru, Venezuela, and Brazil), which represented a lower estimate of 105,000 jaguars or a higher estimate of 307,000 jaguars. Taking jaguar densities such as one jaguar per 15 km^2^ for certain areas of Belize, one jaguar per 33 km^2^ in the Cockscomb Basin, Belize and one jaguar per 25–75 km^2^ in the Paraguayan Chaco region [47,48,49], and applying them to the approximately six million km^2^ of the Amazon, this would yield a census size for Amazonian jaguars ranging from 182,000 to 400,000 individuals. Jaguar population sizes estimated using microsatellite genetic diversity were found to fall squarely within this range. This study demonstrated for the first time the usefulness of microsatellite markers for inferring jaguar population sizes in different regions. **(2)**- The second study conducted in Colombia, which focused primarily on jaguars from that country, was that of Ruiz-García et al. [20], who used the analysis of 49 jaguars with known geographic origins through 18 nuclear microsatellites. In this study, their genetic structure was compared with that of another large Neotropical carnivore (the spectacled bear, *Tremarctos ornatus*). The feline showed substantially higher levels of genetic diversity than the bear, with the levels of genetic heterogeneity among jaguar populations being 10 to 20 times lower than the levels of genetic heterogeneity found among spectacled bear populations. Furthermore, the probability of finding the same multi-genotypic profile between two jaguars sampled in Colombia for these 18 microsatellites was 2.36 × 10^−15^, meaning that no two jaguars in Colombia (or anywhere else in their geographic range) possess the same genetic profile with this collection of microsatellite markers. This result is crucial because it demonstrates that the use of microsatellites allows for the individual identification of each jaguar, which can be leveraged in forensic studies, individualization in reports of attacks on livestock or humans, population density estimates by identifying individual jaguars, social structure, kinship relationships, connectivity between different regions, and migration patterns. **(3)**- Ruiz-García et al. [50] analyzed 62 Colombian jaguars for 12 microsatellites belonging to the two supposed subspecies present in the country (trans-Andean population, *P. onca centralis* n = 15, and cis-Andean population, *P. onca onca*, n = 47). Both the overall sample of jaguars in Colombia (H_e_ = 0.835 ± 0.075 and n_A_ = 10.083 ± 2.571), and the samples belonging to the two putative subspecies (*P. onca centralis*, H_e_ = 0.828 ± 0.083 and n_A_ = 6.250 ± 1.581; *P. onca onca*, H_e_ = 0.826 ± 0.117 and n_A_ = 7.714 ± 2.429) showed high levels of genetic diversity. Although some microsatellites showed significant heterogeneity between the two putative subspecies (*Fca 96*, *Fca 45*, *Fca 391*, and the overall set of 12 microsatellites), the overall F_ST_ value (=0.01–0.02) was extremely low, indicating the absence of molecularly differentiated subspecies in Colombia. Long-term historical estimates for the Colombian jaguar population were estimated with the Nielsen’s [51] procedure (MISAT program) and they ranged from 9755 to 21,851 individuals (θ = 4N_e_μ, unistep mutation model, mutation rate per generation: 5.6 × 10^−4^ and 2.5 × 10^−4^, respectively) and 10,752 to 24,084 individuals (with a multi-step mutation model and the same mutation rates). In general, no signs of potential population bottlenecks affecting the jaguar in Colombia were detected, particularly in the cis-Andean population. However, two analyses detected slight evidence of a possible recent bottleneck in the trans-Andean population (*P. onca centralis*). **(4)**- Ruiz-García et al. [52] expanded this analysis, using 12 microsatellites, to 107 jaguar specimens from the Colombian, Peruvian, and Bolivian Amazon. Five of these 12 microsatellites showed significant genetic heterogeneity, although overall genetic heterogeneity was extremely low (F_ST_ = 0.017), with a high gene flow estimate, regardless of the flow models considered (Nm = 6.42–14.45). When all these Amazonian samples were taken together, significant population expansion was observed. Therefore, this study anticipated that of Lorenzana et al. [41] in demonstrating the existence of a single gene pool for jaguars across much of the Amazon basin. **(5)**- Subsequently, Ruiz-García et al. [53] analyzed 250 jaguars from eight different countries, 156 of which were of Colombian origin, for 12 nuclear microsatellites and three mitochondrial genes (*ND5*, *16S rRNA*, *ATP8*). The highest levels of genetic diversity were found in the western Amazon for both types of markers (nuclear and mitochondrial), indicating the origin of the current jaguar in that area of the Amazon, while the populations of eastern Brazil and northern Mesoamerica showed the lowest genetic diversity, a result that anticipated the findings later reported by Lorenzana et al. [45] using metagenomic data. For example, for microsatellites, the basically Amazonian populations of Colombia (H_e_ = 0.867 ± 0.059, and n_A_ = 13.0 ± 2.522), Peru (H_e_ = 0.883 ± 0.045, and n_A_ = 8.083 ± 1.443) and Bolivia (H_e_ = 0.883 ± 0.043, and n_A_ = 8.75 ± 2.006) showed significantly higher values than the jaguar population of Guatemala (H_e_ = 0.550 ± 0.034, and n_A_ = 1.428 ± 0.787) or the Eastern Brazilian Amazon (Amazon mouth) (H_e_ = 0.742 ± 0.123, and n_A_ = 4.4 ± 1.776). No “true” subspecies were detected in South America at the molecular level, and the Amazon River did not constitute any type of geographic barrier to jaguar dispersal, contrary to what Eizirik et al. [16] had previously claimed. Various AMOVA analyses showed that most of the genetic diversity in jaguars resided within individuals rather than in higher-ranking hierarchical groups. The percentage of first-generation migration for jaguars was high (10–20% of individuals), demonstrating the importance of gene flow in this species. Analysis of the mt*ND5* gene revealed several highly significant population expansions from the western Amazon during three different periods (600,000–500,000 years ago, 250,000–150,000 years ago, and 55,000–30,000 years ago). Ruiz-García [54] also provided new data regarding the absence of molecular subspecies in the jaguar, as well as clear evidence of a strong population expansion for this species during the Pleistocene and the absence of population bottlenecks in northwestern South America. **(6)-** Jiménez-González et al. [55] constructed the pedigrees of 20 captive jaguars in Colombian zoos using nine nuclear microsatellites and the regional Studbook for this species. The genetic diversity of these captive specimens (H_e_ = 0.684 ± 0.230) was compared with that found in the Colombian wild population (n = 156), with an estimated value of H_e_ = 0.867 ± 0.059. Therefore, the Colombian captive jaguar population retained approximately 78% of the genetic diversity found in a large wild jaguar sample. Similarly, the Colombian captive jaguar population showed an n_A_ value of 5.67 ± 2.86, significantly lower than the estimated n_A_ value of 13 ± 2.52 for the wild jaguar sample. **(7)-** Ruiz-García et al. [56] analyzed 157 jaguars for four mitochondrial genes, 73 of which were of Colombian origin. Mitochondrial genetic diversity was very high (H_d_ = 0.995 ± 0.002, and π = 0.0354 ± 0.003), as previously shown in earlier studies. The initial temporal mitochondrial diversification was estimated to have occurred between 1.69 and 1.44 million years ago. A significant spatial structure was found due to the differentiation of specimens from northern Mesoamerica and the southern portion of the continent (Paraguay) compared to most specimens from South America (Mantel test and spatial autocorrelation). However, this spatial structure is moderate, as the Mantel test showed that geographic distances significantly explained only 1.7% of the genetic distances. The spatial autocorrelation analysis showed a moderate, though significant (*p* < 0.0038) pattern of isolation by distance over the more than 4500 km covered by the analysis. The “mismatch distribution” method [57] detected a population expansion for the jaguar that would have originated between 175,000 and 131,000 years ago, while the “Bayesian Skyline Plot” (BSP) procedure [58] detected a strong population expansion for the big cat that would have originated approximately 150,000 years ago and would have stopped about 30,000 years ago. **(8)**- Finally, Ruiz-García et al. (unpublished) analyzed three molecular databases (one with 157 jaguars analyzed for six mitochondrial genes, another with 78 jaguars analyzed for their complete mitogenomes, and another with 112 jaguars analyzed for 18 nuclear microsatellites), with most of the specimens originating from northwestern South America (Colombia, Ecuador, Peru, and Bolivia). Eighteen and nine small clusters were detected in the first two databases, respectively, but these did not have much geographical significance. Geneland’s analysis detected seven genetically distinct populations for mitogenomes and four different populations for microsatellites, but these were mixed, in many cases, in overlapping geographic areas. For example, many small clusters of *P. onca onca* and *P. onca centralis* were found intermingled, with certain groups of *P. onca centralis* more closely related to *P. onca onca* specimens and vice versa. The most peripheral specimens sampled in Guatemala and southern Bolivia and Paraguay were the most genetically distinct, which could correspond to two putative morphological subspecies (*P. onca goldmani* and *P. onca paraguensis*, respectively). As previously determined, mitochondrial and nuclear genetic diversity levels were particularly high in the jaguar population of northwestern South America, consistent with the fact that this geographic area is the original dispersal focus of the current jaguar. Mitogenomic and microsatellite analyses did not detect any evidence of significant spatial genetic structure in northwestern South America. However, using the database of six mitochondrial genes that included animals from Guatemala and southern Bolivia and Paraguay, a significant spatial structure was detected, identical to that was previously reported [56]. Using mitogenomic data, a strong population expansion was detected between 600,000 and 140,000 years ago, peaking around 300,000 years ago, consistent with Eizirik et al. [16]. However, microsatellites detected a sharp population decline in northwestern South America over the last 50,000–30,000 years, coinciding with the findings reported by Lorenzana et al. [45]. In fact, a Msvar analysis [59,60] detected a significant population decline for jaguars in this region of South America over the last 3000 years.

It is interesting to note that when comparing estimates of expected heterozygosity (H_e_), accurate comparisons can only be made if the same microsatellite loci are used. Very few of the cited studies use the same microsatellite loci. However, a thorough analysis of comparative studies shows that H_e_ estimates for different geographic regions are consistent regardless of the microsatellites used. All H_e_ estimates for jaguar populations in Amazonian countries are the highest [30,41,50,52,53], and the estimates of genetic diversity for the Brazilian Pantanal are high, although not as high as those for the Amazon [18,24,25,30] while all estimates for Mexico or Central America are intermediate (more peripatric populations and further from the central range of the Amazon) [27,28,29,36,39], while those for the Brazilian Atlantic Forest always presented the lowest H_e_ estimates due to the extreme fragmentation of that habitat and the small effective numbers of jaguar populations in the Brazilian Atlantic Forest [23,38].

Additionally, the studies of Roques et al. [30] (Amazon and Mexico) and Wultsch et al. [27,28] (Mexico, Belize and Central America), which were carried out with DNA extracted from fecal samples, showed levels of genetic diversity very similar to those obtained with other tissues in the same geographic areas [29,30,36,39,41,50,52,53], and therefore no bias was observed in these studies with respect to the others.

As can be seen, there is a substantial body of genetic research on the jaguar, both for its general distribution in the Neotropics and, specifically, for its distribution in Colombia. In fact, of the 27 molecular studies cited that included jaguars, samples from Colombian jaguars played a primary role in nine of them (33%). Table 1 compares the estimates of different genetic-population parameters found in the Colombian jaguar population with those of other jaguar populations analyzed in other areas of the Neotropics. In general, the jaguar in Colombia shows high values for these parameters and appears to be at less risk, from a genetic perspective, than jaguars in other areas such as Central America and the Brazilian Atlantic Forest, especially.

### 2.2. Puma

The puma (*Puma concolor*) is the second largest feline in the Americas (weighing between 34 and 100 kg and measuring between 0.95 and 1.43 m in head and body length, excluding the tail). Globally, *P. concolor* is listed as Least Concern. The current population trend is decreasing, and the population is not severely fragmented.

Up to 30 subspecies of puma have been proposed in the Americas [61,62]. For the Neotropics, the following subspecies have traditionally been recognized (basically from north to south):

(1) *Puma concolor improcera* (Phillips, 1912). Type locality: Calmalli, Baja California, Mexico.

(2) *Puma concolor azteca* (Merriam, 1901). Type locality: near Casas Grandes, Chihuahua, Mexico.

(3) *Puma concolor stanleyana* (Goldman, 1936). Type locality: Bruni, Webb Co., Texas, USA, but an important extension of its distribution covers northeastern Mexico.

(4) *Puma concolor mayensis* (Nelson and Goldman, 1929). Type locality: La Libertad, Petén department, Guatemala.

(5) *Puma concolor costaricensis* (Merriam, 1901). Type locality: Boquete, Chiriquí, Panama.

(6) *Puma concolor bangsi* (Merriam, 1901). Type locality: Dibulla, Magdalena department, Colombia.

(7) *Puma concolor soderstromii* (Lönnberg 1913). Type locality: Pichincha province, Ecuador.

(8) *Puma concolor concolor* (Linnaeus, 1771). Type locality: Cayenne, French Guiana.

(9) *Puma concolor anthonyi* (Nelson and Goldman, 1931). Type locality: Monte Duida, Amazonas state, Venezuela.

(10) *Puma concolor incarum* (Nelson and Goldman, 1929). Type locality: Piscocucho, Urabamba River, Cuzco department, Peru.

(11) *Puma concolor borbensis* (Nelson and Goldman, 1933). Type locality: Borba, Madeira River, Amazonas state, Brazil.

(12) *Puma concolor osgoodi* (Nelson and Goldman, 1929). Type locality: Buena Vista, Santa Cruz department, Bolivia.

(13) *Puma concolor acrocodia* (Goldman, 1943). Type locality: Descalvados Matto Grosso du Sul state, Brazil.

(14) *Puma concolor greeni* (Nelson and Goldman, 1931). Type locality: Curraes Novos, Rio Grande do Norte state, Brazil.

(15) *Puma concolor capricornensis* (Nelson and Goldman, 1929). Type locality: Piracicaba, Sao Paulo state, Brazil.

(16) *Puma concolor cabrerae* (Pocock, 1940). Type locality: La Rioja, Rioja province, Argentina.

(17) *Puma concolor puma* (Molina, 1782). Type locality: near to Santiago, Chile.

(18) *Puma concolor araucanus* (Osgood, 1943). Type locality: Sierra de Nahuelbuta, Malleco province, Chile.

(19) *Puma concolor patagonica* (Merriam, 1901). Type locality: Santa Cruz province, Argentina and al covers part of Chile.

(20) *Puma concolor pearsoni* (Thomas, 1901). Type locality: Santa Cruz province, Argentina.

Molecular studies of Neotropical puma populations are more limited than those of jaguars. The main studies involving Neotropical pumas were the following: **(1)**- The pioneering work studying North American and South American puma populations was that of Culver et al. [63]. They analyzed 315 pumas, none of Colombian origin, representative of the 30 putative morphological subspecies of the Americas using three mitochondrial genes (*ND5*, *16S rRNA*, *ATP8*) and 10 nuclear microsatellites. Six genetically distinct populations were detected. The North American population proved to be quite homogeneous and with less genetic variability than the Central and South American populations [H_e_ = 0.42 ± 0.016 (microsatellites) and π = 0.0002 (mtDNA) for the North American population; H_e_ = 0.63 ± 0.011 and π = 0.004 for the Central American population; H_e_ = 0.71 ± 0.033 and π = 0.003 for the overall South America population; in the South American population, four different groupings would be distinguished, whose H_e_ and π values would range between 0.64 ± 0.116 and π = 0.0019 (southern South America), 0.71 ± 0.009 and π = 0.0022 (eastern South America), 0.75 ± 0.052 and π = 0.0004 (northern South America), and 0.75 ± 0.046 and π = 0.001 (central South America)]. The North American population would correspond to the subspecies *P. c. couguar* and would have derived in the last 10,000 years from a small number of specimens from one of the South American populations originating from the eastern part of South America, which, in turn, would have formed 200,000–300,000 years ago. The recolonization of the puma in North America from South America (previously, a distinct puma population existed in North America) coincided with the mass extinction of large vertebrates in that subcontinent at the end of the Pleistocene. In Central America, a single population (*P. c. costaricensis*) was also detected. In South America, four different populations were identified: one in the northern and northwestern part of South America (*P. c. concolor*), another in the eastern part of South America (*P. c. capricornensis*), another in the central part of South America (*P. c. cabrerae*), and the last in the southern and southwestern part of South America (*P. c. puma*). **(2)**- Ruiz-García [46] analyzed 50 pumas (30 from Colombia, specifically the northern coast and Amazon region, 10 from the Loreto Department in the Peruvian Amazon, and 10 from the Santa Cruz Department in Bolivia) for five microsatellite loci. Genetic diversity was high (H_e_ = 0.749 ± 0.243, n_A_ = 7.40 ± 2.10), like that reported by Moreno et al. [17] for 18 pumas from Brazilian zoos analyzed for four microsatellite loci (PIC = 0.663 ± 0.167 and n_A_ = 9.25 ± 2.86). Pumas of Colombian origin showed some evidence of a bottleneck (standardized difference test, *p* = 0.039; Wilcoxon test, *p* = 0.0156 for the IAM model), while no evidence of a bottleneck was detected in the Peruvian and Bolivian samples. When analyzed together, the three geographic areas showed some positive evidence of a bottleneck (standardized difference test, *p* = 0.033; Wilcoxon test, *p* = 0.0102 for the IAM model). Historical estimates of N_e_ for pumas ranged between 74,600 and 185,900 animals (they could represent at census level between 88,200–287,800 animals). **(3)**- Ruiz-García et al. [64] analyzed eight pumas from the Bolivian highlands (five specimens differentiated from 25 fecal samples collected in the Sajama National Park, Oruro department, and three skin samples, one from the south of the La Paz department and two from the Cochabamba department) that “a priori” would belong to the presumed morphological subspecies *P. c. osgoodi*. These samples were compared with 45 other puma samples from other geographic areas of northwestern South America [Colombia (departments of Atlántico, Bolívar, Risaralda, Valle del Cauca, Vaupés, Vichada, and Amazonas; a total of 33 Colombian pumas), Ecuador, Peru, Venezuela, and the western part of the Brazilian Amazon] that presumably belonged to other putative morphological subspecies of puma (*P. c. anthonyi*, *P. c. bangsi*, *P. c. soderstromi*, and *P. c. incarum*) using seven nuclear microsatellites. The genetic diversity for the two puma groups was very high (H_e_ = 0.942 ± 0.107 for the Bolivian population; H_e_ = 0.845 ± 0.091 for the remaining pumas). Conversely, the Bolivian sample value for n_A_ = 3.86 ± 1.46 was significantly lower than the value for the other countries’ samples, n_A_ = 11 ± 3.92; however, n_A_ is strongly influenced by sample size (eight specimens versus 45). A multigenotype assignment analysis correctly classified only between 52.8% and 79.24% of the pumas studied. A significant number of Bolivian pumas were assigned to the other geographic group. A UPGMA tree with shared allele distance (DAS) showed that Bolivian pumas were distributed in different groups with other pumas from different geographic areas. The *Fca 96* marker exhibited the greatest significant genetic heterogeneity among the pumas from the various countries studied. For the most part, although some global and individual statistics were significant (F_ST_ = 0.011; R_ST_ = 0.049–0.069), the genetic differences were small, so it was concluded that all the pumas analyzed in that northwestern area of South America belong to a single genetic pool, largely coinciding with what was found by Culver et al. [63]. At the beginning of 2010, several studies emerged analyzing the genetic structure of pumas in southern Brazil, an area heavily impacted by human activity and with significant habitat fragmentation. **(4)**- Castilho et al. [65,66] analyzed 37 individuals in this region (states of Rio Grande do Sul, Santa Catarina, and Paraná) using 18 microsatellites. This population showed evidence of having recently experienced a bottleneck with a loss of genetic variability, although it still maintains moderately high genetic diversity (H_e_ = 0.682 ± 0.173, n_A_ = 5.888 ± 1.791). Different analyses (PCAGEN, GENECLASS, STRUCTURE) detected a single gene pool in the area. The ONESAMP 1.1 program estimated the effective population size at 23.5 (95% confidence interval: 20.74–31.5). Despite intense habitat destruction and fragmentation, connectivity and gene flow were determined among pumas in this geographic area. **(5)**- Miotto et al. [67] analyzed 111 samples (mostly scat) from pumas in different areas of the Brazilian state of São Paulo using seven nuclear microsatellites. Genetic diversity was high (H_e_ = 0.797 ± 0.039, n_A_ = 9.286 ± 1.906), with no evidence of inbreeding (F_IS_ = −0.022; *p* = 0.0083), but rather of outbreeding. The STRUCTURE program detected a single population, and a recent bottleneck was also identified within that population. The estimated effective population size using the LDNe program was 39.2 animals. **(6)**- However, Saranholi et al. [68] detected significant genetic differences between samples of 16 pumas analyzed at two different points along the Tietê River in the Brazilian state of São Paulo (dos Caetetus EE, n = 6; Itirapina EC, n = 10) for seven microsatellites. STRUCTURE analysis determined the existence of two genetically distinct populations. Similarly, Factorial Component Analysis (FCA), Geneland, and F_ST_ analyses (=0.082, *p* < 0.01) detected significant genetic differentiation between these two populations. The presence of the Tietê River may have generated the differentiation between the two populations at a microgeographic scale. However, genetic diversity in both locations was high and similar (for Caetetus EE: H_e_ = 0.77 ± 0.089 and n_A_ = 7 ± 1.414; Itirapina EE: H_e_ = 0.783 ± 0.073 and n_A_ = 5.714 ± 1.666, respectively). This work provided evidence, for the first time, of some genetic differentiation within *P. c. capricornensis*. A Migrate-N gene flow analysis indicated a limited and preferential gene flow from Caetetus EE to Itirapina EE, which may reflect past or remnant gene flow between both areas. **(7)**- Caragiulo et al. [69] analyzed 601 puma DNA samples (many from scat; no samples of Colombian origin) using four mitochondrial genes (*Cyt-b*, *12S rRNA*, *16S rRNA*, *ATP6*). Only 160 samples were amplified for all four genes. They agreed with the study by Culver et al., [63] in that they differentiated three genetic clusters (North America, Central America, and South America), with the latter two, especially the third, (five and 11 haplotypes, respectively, and π = 0.0017 and 0.0022, respectively) exhibiting much greater genetic diversity than the first cluster (two haplotypes and π = 0.0006), concluding that the homogeneous North American population originated from the South American puma population. The Central American population, although distinct, presented haplotypes shared with the South American population and with the North American population. **(8)-** Matte et al. [70] analyzed 186 pumas (156 for South America, 17 for Central America and 13 for North America) for the mt*ND5* gene. These authors detected seven genetically distinct geographic groupings of pumas: northern Central America + North America (H_d_ = 0.496 ± 0.119, π = 0.0018 ± 0.0009, two haplotypes), southern Central America (H_d_ = 0.733 ± 0.155, π = 0.0061 ± 0.0015, three haplotypes), northern South America (H_d_ = 0.333 ± 0.215, π = 0.0011 ± 0.0007, two haplotypes), central-northern-eastern South America (H_d_ = 0.828 ± 0.027, π = 0.0032 ± 0.0003, 11 haplotypes), eastern South America (H_d_ = 0, π = 0, one haplotype), south-central South America (H_d_ = 0.810 ± 0.078, π = 0.0025 ± 0.0005, three haplotypes), south-western South America (H_d_ = 0.600 ± 0.073, π = 0.0033 ± 0.0003, three haplotypes). Clearly, the grouping that presented the greatest genetic diversity was that of north-central-eastern South America, although it also had by far the largest sample size. When genetic diversity statistics were analyzed by subcontinent, the same general pattern was observed, with the North American population having much lower genetic variability than the global South American population (North America: H_d_ = 0.259 ± 0.156, π = 0.0022 ± 0.0016, two haplotypes; Central America: H_d_ = 0.794 ± 0.075, π = 0.0052 ± 0.0011, five haplotypes; South America: H_d_ = 0.904 ± 0.011, π = 0.0043 ± 0.0003, 22 haplotypes). Haplotype H9 was found to be shared between some pumas from Costa Rica (southern Central America) and some from South America, and haplotype H23 is shared by pumas from Central America and North America. In the haplotype network, the haplotype closest to the outgroup (a *Puma yagouaroundi*) was found in pumas from Paraguay and several states in south-central Brazil, supporting this region of South America as the origin of the modern puma. This study also observed that, globally for all the pumas studied, the Mantel test (r = 0.48; *p* < 0.001) and spatial autocorrelation showed a significant correlation between genetic and geographic distances. However, using data exclusively from South American pumas, no significant influence of isolation by distance was found. Various analyses showed, for both the total sample and the South American sample, the existence of a recent population expansion. The BSP procedure revealed that this expansion occurred in the last 8000 years in South America, meaning that the recolonization of North America by the puma occurred after this period. **(9)**- Miotto et al. [71], using non-invasive samples (faecal DNA), were the first to use this procedure to determine puma abundance in two protected areas embedded in a human-disturbed landscape in the northeast of São Paulo state (Brazil). In eight months of mark-recapture feces sampling, 15 individual pumas were identified using seven microsatellite loci. The estimated abundance of pumas with the Jolly-Seber open population model was 23.81 ± 6.22. This was the first estimate of puma abundance in a human-dominated landscape in São Paulo state, the most populous, developed, and industrialized state in Brazil. It is well documented that genotyping fecal DNA using microsatellites suffers from high rates of allelic dropout and thus underestimates measures of diversity. However, many of the cited studies have incorporated measures to try to minimize the effects of allelic dropout. Some studies that used fecal samples [64,67,71] showed the same levels of genetic diversity as other studies carried out with pumas in the same geographical areas and with different sets of microsatellites [63,65,66,68,70,72]. **(10)**- We must not forget several works previously mentioned [28,36,44], which also provided important results for Neotropical pumas and were cited when discussing jaguars. More recently, new research has emerged involving the South American puma. **(11)**- Saremi et al. [72] analyzed the complete genome of 10 pumas (two Brazilian and eight North American). The most relevant results of this interesting study showed that the North American population originated from the South American puma lineage between 300,000 and 100,000 years ago (estimates much older than in previous studies). The North American populations showed evidence of strong inbreeding, but of a different nature in each population, suggesting that if gene flow between these populations were restored, local levels of genetic diversity could be re-established. Additionally, it was detected that the genome of a Florida puma descends from translocated Central American pumas, but, despite this recent interbreeding, there are still large areas of the genome in this puma that show evidence of homozygosity from previous inbreeding processes, which highlights that multiple translocations must be carried out or connectivity from other areas with Florida pumas must be increased in order to erase the persistent effects of inbreeding in their genome. **(12)**- Gallo et al. [73,74] analyzed 83 pumas from three south-central provinces of Argentina (Buenos Aires, Río Negro, and Chubut) for 25 nuclear microsatellites (Buenos Aires: H_e_ = 0.655 ± 0.185 and n_A_ = 5.4 ± 1.8; Río Negro: H_e_ = 0.706 ± 0.142 and n_A_ = 5.8 ± 1.8; Chubut: H_e_ = 0.704 ± 0.114 and n_A_ = 5.6 ± 1.9; global sample: H_e_ = 0.713 ± 0.134 and n_A_ = 6.9 ± 2.4). Therefore, the genetic diversity for these Argentine pumas can be considered moderately high. A STRUCTURE analysis detected two genetically distinct puma populations in the three Argentine provinces studied (Buenos Aires and Río Negro on one hand, and Chubut on the other). Gene flow was low between the two groups and asymmetric, with gene flow from Buenos Aires-Río Negro to Chubut being five times greater than in the reverse case. Both groups showed evidence of recent bottlenecks, but these were more pronounced in the Chubut group. As with the jaguar, many of the studies conducted with pumas have used different collections of microsatellites. For instance, Moreno et al., [17] used 4 microsatellites from domestic cats, meanwhile Culver et al., [63] used 10 loci from domestic cats (and only two overlapped with [17]), and Castilho et al., [65,66] used 18 loci, 14 of which were developed specifically for pumas. Nevertheless, in all the cases, the H_e_ values for South American puma populations were the highest, while H_e_’s estimates in Central America and Mexico are always intermediate and those in North America are always the lowest, regardless of the set of microsatellite loci used. **(13)**- Ruiz-García et al. [56] analyzed 177 pumas from 11 Latin American countries for four mitochondrial genes. Of these, 67 specimens were from Colombia. Overall, the genetic diversity found for this feline species was very high (H_d_ = 0.95 ± 0.013; π = 0.036 ± 0.003), generally coinciding with findings in other studies for South American puma populations. The onset of temporal diversification of mitochondrial haplotypes in pumas was estimated to have occurred between 1.87 and 1.64 million years ago. A significant spatial structure was detected when considering pumas sampled in Central and South America, consistent with previous findings [68]. The Mantel test showed r = 0.21 (*p* < 0.0001), indicating that geographic distances significantly explained 4.2% of the genetic distances. Similarly, the spatial autocorrelation for these pumas was highly significant (*p* < 0.0003), with a very pronounced cline within the first 2000 km, but with no spatial structure between 2000 and 7000 km. This spatial structure was primarily driven by differences between specimens sampled in Central and South America, and especially between populations in northern South America and different populations in southern South America. As in other previously cited studies for this global group of pumas, strong evidence of a significant population expansion was detected. The mismatch distribution procedure detected a population expansion between 62,000 and 46,000 years ago, while the BSP procedure detected it between 300,000 and 250,000 years ago, coinciding with the findings of the genomic study by Saremi et al. [72]. This procedure also detected a population decline in the female lineages about 15,000 years ago. **(14)**- Finally, Mac Allister et al. [75] analyzed 180 pumas from south-central Argentina for two mitochondrial markers (control region and *ND5*). Samples from central Argentina (northern Buenos Aires, southern Córdoba and Santa Fe provinces) showed high genetic diversity (H_d_ = 0.806 ± 0.079; π = 0.0048 ± 0.0013), while the population sampled further south, in Patagonia, showed considerably lower genetic diversity (H_d_ = 0.163 ± 0.054; π = 0.0003 ± 0.0006). The intermediate population between these two cited populations showed similar genetic diversity values to the first population (H_d_ = 0.820 ± 0.042; π = 0.0040 ± 0.0010). Bayesian phylogenetic trees identified two main groupings. One group corresponded to specimens from Patagonia, and the other to the rest of the studied area in Argentina. The temporal separation between these two groupings was estimated at approximately 228,000 years ago. Mitochondrial diversification within each grouping was estimated at 46,000 years ago (Patagonia) and 172,000 years ago (central and intermediate areas). A BAPS analysis detected the existence of three distinct groupings: the one already mentioned in Patagonia and two distinct populations in central Argentina. The Patagonian population showed evidence of a very strong population expansion (greater than that of the central Argentine population).

Unlike the situation with the jaguar, where numerous studies have included DNA samples of Colombian origin and some have focused on describing the jaguar’s genetic structure in Colombia, only three studies published on the puma have included DNA samples of Colombian origin, and none of these primarily focused on describing the puma’s genetic structure in Colombia. Furthermore, two of these three studies can be considered outdated. In fact, the percentage of studies that incorporated samples from Colombian pumas, when analyzing the 17 studies referenced here for Neotropical pumas, was much lower than in the case of the jaguar mentioned above (3/17 = 17.6% versus 9/27 = 33.3%). Therefore, a specific and up-to-date study is recommended to gain a comprehensive understanding of the different genetic and population parameters of the puma in Colombia. A comparison of some of these parameters between Colombian pumas and those from other areas of the Neotropics can be seen in Table 2. In general, fewer genetic studies have been published on pumas in the Neotropics than on jaguars. This statement does not consider the numerous studies of this kind that have been published on pumas in North America [76,77,78,79,80,81].

### 2.3. Jaguarundi

The jaguarundi (*Puma = (Herpailurus) yagouaroundi*), phylogenetically related to the puma and the cheetah (*Acynonyx jubatus*), is a felid much smaller than those just mentioned (3.5–7 kg in weight and head and body length, excluding the tail, between 50 and 70 cm). Globally, *P. yagouaroundi* is listed as Least Concern. The current population trend is decreasing, and the population is not severely fragmented.

Cabrera [82] defined eight subspecies for the jaguarundi, and subsequently all authors adopted this classification scheme [83,84,85]:

(1) *P. y. cacomitli* (Allen, 1919) for the animals from southern Texas, USA (Cameron, Hidalgo, Starr and Wilacy Counties) and along the eastern coast of Mexico (Tampico and Tamaulipas) until Veracruz.

(2) *P. y. tolteca* (Thomas, 1898) was separated from the previous putative subspecies. Its distribution ranges from southern Arizona in USA (Cochise, Pima and Santa Cruz Counties) and southwards in a narrow strip along the Pacific coast of Mexico to Sinaloa and Guerrero state.

(3) *P. y. fossata* (Mearns, 1901) was also separated from *P. y. cacomitli*. Its distribution ranges south and east from Oxaca to Veracruz in Mexico and in Guatemala (in the Peten, Izabal, Progreso, Mazatenango, and Suchitepequez departments) as well as in El Salvador, Honduras, Belize, and Nicaragua.

(4) *P. y. panamensis* (Allen 1904) is distributed in Costa Rica, Panama, northern and western Colombian and western Ecuador.

(5) *P. y. yagouaroundi* (Geoffroy, 1803) is the *P. y. unicolor* defined by Allen [86]. Its distribution includes eastern Venezuela, Guianas and northeastern Brazil.

(6) *P. y. melantho* (Allen, 1919), has a distribution range that includes Andean valleys from Peru in La Libertad, Huánuco, Pasco, Junín, and Puno departments as well as a broad distribution in the western Amazonian (Loreto, Ucayali and Madre de Dios departments).

(7) *P. y. eyra* (Fischer, 1814) is the *P. y. jaguarundi* of Allen [86] has a geographical distribution ranging from southern Brazil, including São Paulo state, Paraguay, northeastern Argentina in the Misiones, Mesopotamia and Chaco regions, extending south at least as far as northern Entre Ríos and northeastern Córdoba provinces and west to western Salta and Tucumán provinces. The jaguarundi found in Bolivia could also be *P. y. eyra* [87,88].

(8) *P. y. ameghinoi* (Holmberg, 1898) is distributed in the sub-Andean low mountain zone of western Argentina, from Jujuy south to northern Patagonia (west of Viedma at 41°S).

The jaguarundi is one of the Neotropical feline species for which the fewest population genetic-molecular studies have been conducted. However, four of the seven genetic studies conducted to date involve animals of Colombian origin. **(1)**- In the first study, Ruiz-García [46] determined H_e_ = 0.616 ± 0.38, and n_A_ = 4.6 ± 1.7 for 16 jaguarundis from Colombia (Caribbean coast and Amazonas), Venezuela, Peru (Loreto department, Peruvian Amazon), Bolivia and southern Brazil (Rio de Janeiro state) at the microsatellites *Fca08*, *Fca43*, *Fca45*, *Fca96* and *Fca126*. These levels of genetic diversity were moderate, although that first sample analyzed was very small. No population bottlenecks were detected, and the estimated long-term effective numbers ranged from 40,100 to 72,300 individuals, which would imply census numbers between 53,300 and 108,400 individuals if a N_e_/N ratio of 0.5 to 0.67 is used. This species is considered Least Concern by the IUCN and is considered abundant throughout its range. However, it appears to be extinct in Uruguay [89] and seems to have disappeared from Texas (USA). The last documented record in Texas was in 1986. Lombardi et al. [90] conducted a camera-trap survey from 2003 to 2021 across southern Texas and Tamaulipas (Mexico) and, after 350,366 trap nights at 685 camera sites, did not detect any jaguarundis in Texas. The moderate nuclear genetic diversity observed in that study may have been due to the small sample size. However, the protection of this species should not be neglected, as it may be less abundant than expected. **(2)**- Other works that included the jaguarundi were those of Eizirik et al. [22] and Moreno et al. [17], although these were not strictly population-genetic works. The first study [22] analyzed the melanism in cats, jaguars and jaguarundis and determined a deletion in the *MC1R* gene which causes melanism in the jaguarundi, which differed from mutations which cause melanism in cats and jaguars. The second study [17] determined a PIC = 0.825 ± 0.067, and n_A_ = 10.75 ± 3.63 for a group of captive jaguarundis (n = 36) in Brazilian zoos for four microsatellite loci. They concluded that these markers are useful for determining gene diversity levels in jaguarundi, pumas and jaguars in captivity. **(3)**- But the first phylogeographic and population-genetic work, although preliminary, was carried out by Ruiz-García and Pinedo [91], through the analysis of three mitochondrial genes (*ND5*, *16S rRNA*, *ATP8*) applied to 44 jaguarundis [Mexico (n = 1, Jalisco state), Guatemala (n = 1, Petén department), Costa Rica (n = 2, Heredia province), Colombia (n = 10, one from the department of Risaralda, from Córdoba, and from Valle del Cauca “a priori” classified as *P. y. panamensis*; one from the department of Guainía, from Arauca, from Tolima, from Huila, and three from the department of Meta, classified “a priori” as *P. y. melantho*), Venezuela (n = 3, Falcon state and Canama National Park), French Guiana (n = 1), Ecuador (n = 7), Peru (n = 14, Pasco, Huánuco, Junín, San Martín, Ucayali, and Loreto departments), Bolivia (n = 3, La Paz and Santa Cruz departments), and Brazil (n = 2, Rio de Janeiro state)]. For the overall sample, very high values of genetic diversity were obtained (H_d_ = 0.960 ± 0.078; π = 0.055 ± 0.008), which contrasts with what was observed at the nuclear level. Only for Colombia were these values also very high (H_d_ = 0.980 ± 0.091; π = 0.063 ± 0.022). Considering only the mt*ATP8* and mt*16SrRNA* genes, the Bolivian samples differed significantly from the others; However, at the mt*ATP8* gene, no statistics showed significant heterogeneity and the relative gene heterogeneity statistics were all very small (G_ST_ = 0.0329, N_ST_ = 0.0022 and F_ST_ = −0.0033) and the gene flow estimates were clearly very high (Nm = 5.91–∞). No subspecies pairs showed important genetic heterogeneity levels. The same was found at the mt*16S rRNA* gene. No statistics showed significant heterogeneity and the relative gene heterogeneity statistics were all very small (G_ST_ = 0.0515, N_ST_ = 0.013 and F_ST_ = 0.016) and the gene flow estimates were clearly very high (Nm = 4.67–37.29). No morphological subspecies pairs showed important genetic heterogeneity levels. Two AMOVAs applied at the mt*ATP8* gene showed that more than 95% of the genetic variance was among the individuals within the countries, only around 16% of genetic variance among countries and negative genetic variance values (that is, not genetic variance) among the putative morphological subspecies. The two AMOVAs applied at the mt*16S rRNA* gene showed that all the genetic variances found were among individuals within countries and no genetic variance was found among countries or among putative morphological subspecies (both presented negative variances). Thus, this was the first proof that putative morphological subspecies seem to be nonexistent for the jaguarundi. The different groupings found in the phylogenetic trees presented intermixed specimens of different putative subspecies. Some statistics were observed with a sign of population expansion. A BSP analysis using the mt*ATP8* gene detected a strong population expansion in maternal lineages around 400,000 years ago, with a population decline in the last 20,000 years. The same analysis using the mt*16S rRNA* gene also detected a population decline around 20,000 years ago. Many of these results were considered preliminary at the time because the sample size was very limited. **(4)**- Holbrook et al. [92] analyzed 11 jaguarundis captured between 1991 and 2004 in Tamaulipas, Mexico, and analyzed them for 12 nuclear microsatellites. They found low levels of genetic diversity (H_e_ = 0.49 ± 0.22 and n_A_ = 4 ± 1.65). They found a F_IS_ value of 0.11, indicating an 11% excess of homozygotes (typical of inbreeding) in this peripheral jaguarundi population. For a mitochondrial marker (a fragment of the 437 bp control region), they found no genetic variability in this jaguarundi sample because it only detected a single haplotype. However, this result could be an artifact of the very small sample size used. The probability of identifying two different jaguarundis as the same individual was low using only 4–7 microsatellite loci (P(ID) < 0.001 and P(ID)sib < 0.01, respectively). However, they documented diversity at the *MC1R* gene for fur coloration. The frequency of the melanistic mutation (gray phenotype) was 0.33, whereas the frequency was 0.67 for the ancestral allele (red phenotype). Therefore, nuclear diversity of this sample of jaguarundis was less than that of the sympatric population of ocelots (*L. pardalis*) in the same area of Tamaulipas in Mexico; even, mitochondrial diversity was much lower. The frequency of the melanistic mutation was lower in this sample than previously reported in zoo jaguarundis. **(5)**- Ruiz-García et al. [93] analyzed 80 jaguarundis for their complete mitogenomes. Of these, 21 specimens were of Colombian origin (one from the department of Guajira, three from the department of Cesar, one from the department of Córdoba, one from the department of Sucre, one from the department of Bolívar, one from the department of Chocó, one from the department of Risaralda, one from the department of Tolima, one from the department of Huila, one from Cauca, all of them “a priori” *P. y. panamensis*, plus one from the department of Arauca, one from the department of Vichada, one from the department of Guainía, plus six specimens from the department of Meta, all of them “a priori” *P. y. melantho*). The mitogenomes of the 80 jaguarundis showed, in a maximum likelihood phylogenetic tree, the existence of at least five different groupings. One group has a wide distribution due to the species’ range (Mexico, Guatemala, Costa Rica, Panama, Colombia, Venezuela, Ecuador, Peru, French Guiana, Brazil, Bolivia, Paraguay, and Argentina), while other groups correspond to very specific geographic areas. These are: (1) a group in Bolivia, (2) a group in the Chaco region of Paraguay and Argentina (plus the province of Misiones), (3) a group in the Colombian Eastern Llanos, (4) a group in the department of Cesar (northern Colombia), and (5) a group that extends from Costa Rica to the Pacific coast of Ecuador, including trans-Andean areas of Colombia. Similarly, an analysis with Geneland also detected five distinct populations: (1) a population in the Paraguayan Chaco and northern Argentina, (2) the aforementioned population of Cesar (northern Colombia), (3) a population in the central and southern area of the Peruvian Andes, (4) a population that extends from Costa Rica through certain areas of northern Colombia, the Eastern Llanos and the Ecuadorian Pacific coast, and (5) the population that extends over most of the jaguarundi’s geographical distribution. Both the overall sample (H_d_ = 0.995 ± 0.001 and π = 0.0472 ± 0.0002) and each of the distinct geographic groupings showed very high levels of genetic diversity (e.g., the Argentine and Paraguayan Chaco: H_d_ = 1.000 ± 0.001 and π = 0.0143 ± 0.0001; southern Central America and trans-Andean Colombia and Ecuador: H_d_ = 1.000 ± 0.001 and π = 0.0733 ± 0.0002). The spatial genetic structure of the jaguarundi was very striking. The overall correlogram (spatial autocorrelation) was not significant, but certain very distant populations (e.g., the Colombian grouping in Cesar and the population in the Paraguayan and Argentine Chaco) were particularly closely related genetically. The onset of mitochondrial diversification in the jaguarundi was estimated to have occurred approximately 3.2 million years ago, earlier than in other Neotropical feline species. Some statisticians, along with the BSP procedure, detected a significant population expansion for jaguarundi that began around 700,000 years ago when complete mitogenomes were used, while this population expansion began 300,000 years ago when only the mitochondrial control region was analyzed. **(6)**- Ruiz-García et al. [56] analyzed the same jaguarundi sample, but only for four mitochondrial genes, to compare these results with those of other Neotropical feline species. Genetic diversity levels were very high (H_d_ = 0.995 ± 0.004; π = 0.0473 ± 0.004), as had been found in previous studies. The initial moment of mitochondrial diversification was estimated at 3.1 million years ago. The Mantel test for the overall sample was not significant (no isolation model by distance). Spatial autocorrelation was also not significant, although populations at very distant distances showed significant similarity. The application of Monmonier’s algorithm [94] detected four distinct populations for this species: (1) one located on the Pacific coast of Ecuador and Colombia (from the province of Esmeraldas in Ecuador to the department of Cauca in Colombia), (2) another located in central Colombia (departments of Tolima and Huila), (3) another located from Risaralda to the Eastern Llanos of Colombia (Arauca and Vichada), and (4) another that extended from the department of Santa Cruz (Bolivia), across Paraguay to the Argentine Chaco. Both the “mismatch distribution” procedure (500,000 years ago) and the BSP procedure (700,000 years ago) detected a significant population expansion for the jaguarundi. Therefore, the results of this latter study were very similar to the one previously discussed using whole mitogenomes. (7) Tamazian et al. [95] assembled the complete genome of a male jaguarundi for the first time. The assembled genome contains a series of scaffolds that reach the length of chromosome arms and is similar in scaffold contiguity to the genome assemblies of cheetah and puma, with a contig N50 = 100.2 kbp and a scaffold N50 = 49.27 Mbp.

Although the number of molecular studies on the jaguarundi is very limited, Colombian specimens are well represented in these studies. In fact, only five population-based genetic studies have been published on this species, and in four of them, Colombian jaguarundis are the subject of study (80%). Table 3 compares the estimates of different population-based genetic parameters in the aforementioned studies. This is a species that requires much more in-depth molecular research, particularly in countries like Brazil, Paraguay, Argentina and Central America where molecular studies of this species are needed at a micro-geographic level.

### 2.4. Ocelot

The ocelot (*Leopardus pardalis*) is the largest of the species of the genus *Leopardus* (8–16 kg in weight and head and body length, not including the tail, between 70 and 100 cm) and ranges from the southern United States to northern Argentina. Globally, *L. pardalis* is listed as Least Concern. The current population trend is decreasing, and population is not severely fragmented.

Murray and Gardner [96], based on diverse authors [82,97,98], proposed the following subspecific classification for the ocelot:

(1) *Leopardus pardalis aequatorialis* (Mearns, 1903) (with *mearnsi* and *minimus* as synonymous). This form basically coincides with the subspecies proposed by Allen [86]. Its distribution is throughout the Peruvian, Equatorian and Colombian Andes to Panama. Although Allen [86] and Pocock [99] recognized this subspecies, the geographical limits for taxon are different for diverse authors. Exemplars from Gualaquiza and Guayas (Ecuador), Marcapata, Yurac Yacu, San Martin, Pozuzo, Santa Ana-Cuzco (Peru) and Caquetá, Bogotá and Medellín (Colombia) were classified within this subspecies.

(2) *Leopardus pardalis albescens* (Pucheran, 1855) (*limitis* and *ludoviciana* as synonymous). It is also equivalent to Allen’s subspecies, *Leopardus pardalis griffithii*. The type locality is Arkansas and for the synonymous subspecies *limitis* it is Brownsville on the Rio Grande, south of Texas. Formerly, from Arkansas, Louisiana, west and south of Texas (USA) to Soto, La Marina in Taumalipas (Mexico).

(3) *Leopardus pardalis melanurus* (Ball, 1844) (*maripensis* and *tumatumari* as synonymous). Its distribution could be Venezuela (Essequibo River), British (Supinaam River and Moon mountains) and Dutch Guianas, Suriname, Takutu River in the Rio Branco state (Brazil) and possibly the Trinidad Island.

(4) *Leopardus pardalis mitis* (Cuvier, 1820) (*armillatus*, *brasiliensis*, *chati*, *chibigouazou*, *chibiguazu*, *hamiltonii*, *maracaya and smithii* as synonymous). Its distribution extends from Rio Grande do Sul (Brazil) and northern Argentina (Misiones, Corrientes and Tucuman provinces), crossing Paraguay until the southern Brazilian Amazon.

(5) *Leopardus pardalis nelsoni* (Goldman, 1925). The geographical distribution of this putative subspecies is from Oxaca to southern Sinaloa in Mexico, with the type locality in Manzanillo, Colima (Mexico).

(6) *Leopardus pardalis pardalis* (Linnaeus, 1758) (*canescens*, *griffithii*, *griseus*, *ocelot*, *pictus*, *buffoni*, *and mexicana* as synonymous). It is the same subspecies determined by Allen [86]. Its distribution includes Veracruz, Guatemala (Dueñas) and is along the southwestern coast of Mexico from Oaxaca to Colima.

(7). *Leopardus pardalis pseudopardalis* (Boitard, 1842) (*sanctaemartae* as synonymous). It has a distribution in northern Colombia and in northern Venezuela. This is equivalent to the subspecies *sanctaemartae* proposed by Allen [86]. However, Pokock [99] explicitly denied the existence of the subspecies *sanctaemartae* proposed by Allen [86].

(8) *Leopardus pardalis pusaeus* (Thomas, 1914). It is the same subspecies determined by Allen [86] and Pocock [99] for western Ecuador. Its type locality is Chongon, coast region of Ecuador, 15 miles west of Guayaquil; it is a small pale form from the southern coast region of Ecuador.

(9) *Leopardus pardalis sonorensis* (Goldman, 1925). Its geographic distribution is from the Sierra Madre and northern Sinaloa (Mexico) to Arizona.

(10) *Leopardus pardalis steinbachi* (Pocock, 1941). The type locality is from Buenavista, Santa Cruz in Bolivia.

**(1)**- The first relevant molecular study of the ocelot was conducted by Eizirik et al. [100], who analyzed 39 ocelots from nine Latin American countries (no Colombian specimens) for the mitochondrial control region marker (*HVS-I*). They identified four phylogeographic groups of ocelots: (1) a group south of the Amazon River (Bolivia and Brazil south of the Amazon River); (2) northwestern South America (Panama, Venezuela, Trinidad Island, and Brazil north of the Amazon River); (3) northeastern South America (French Guiana and the easternmost part of northern Brazil); and (4) Central America. Overall, this sample of ocelots showed high levels of genetic diversity (H_d_ = 0.962 ± 0.015 and π = 0.068 ± 0.034). Each of the four groups also showed high levels of genetic diversity. The two populations that showed the most moderate levels of nucleotide diversity were Central America (π = 0.020 ± 0.012) and southern South America (π = 0.022 ± 0.0012). Based on this work, four subspecies of ocelot could be proposed (*L. pardalis pardalis*, *L. pardalis* ssp., *L. pardalis pseudopardalis*, and *L. pardalis mitis*). **(2)**- The first study to introduce a substantial sample of ocelots of Colombian origin was Ruiz-García [46], who analyzed 68 samples of this species using five nuclear microsatellites. Forty-eight samples were of Colombian origin (primarily Chocó, Eastern Llanos, and Amazonia), and 20 were of Peruvian origin (Peruvian Amazon, Loreto Department). Genetic diversity levels were very high (H_e_ = 0.837 ± 0.103 and n_A_ = 10.00 ± 2.10), consistent with the findings reported years later by Grisolia et al. [101] with 77 ocelots from various Brazilian zoos, analyzed for four microsatellite loci (H_e_ = 0.845 ± 0.036 and n_A_ = 12.50 ± 0.87). Despite these high levels of genetic diversity, some evidence of bottlenecks was detected in some of the analyzed populations. Colombian ocelots showed evidence of a bottleneck in the Wilcoxon test using the IAM model (*p* = 0.0156). When the Colombian and Peruvian ocelot samples were analyzed together, evidence of a recent bottleneck was again detected for the standardized differences test (*p* = 0.011) and the Wilcoxon test (*p* = 0.0156) for the IAM model. Long-term global effective numbers of ocelots ranged from 128,000 to 447,000. Using a N_e_/N ratio of 0.4 (an effective population of 500 ocelots would correspond to approximately 1334 individuals [102]), census numbers would range from 154,000 to 670,000 ocelots. The hunting of ocelots for their fur was very intense between the late 1960s and early 1970s. In the Iquitos region (Loreto department, Peru) alone, some 40,000 pelts were exported annually, and up to 200,000 ocelot pelts were exported annually throughout the Amazon during that period. Despite this, ocelot population sizes appear to be large. In fact, Emmons [103] estimated the existence of about 800,000 ocelots in South America, although she suggested that more realistic figures might be between 1.5 and 3 million. The estimates obtained from molecular data would fall at the lower end of this range. **(3)**- Ruiz-García et al. [52] analyzed 133 ocelots that were classified “a priori” by putative subspecies and were analyzed for 12 nuclear microsatellites [14 samples of *L. p. melanurus* (=*maripensis*) from Colombia, northern Brazil and French Guiana, 49 samples of *L. p. aequatorialis* from various areas of Colombia and Peru, two samples of *L. p. aequatorialis* (=*mearnsi*) from Costa Rica, 42 samples of *L. p. pseudopardalis* all of them basically from northern Colombia and some of Venezuelan origin, 14 samples of *L. p. pusaeus* from the western area of Ecuador, 11 samples of *L. p. steinbachi* from Bolivia, and one sample of *L. p. mitis* from Paraguay]. The genetic diversity found for the total sample was very high (H_e_ = 0.905 ± 0.124 and n_A_ = 17.33 ± 4.21). Genetic diversity estimates were also high for the two putative subspecies assumed to be found in the Colombian Amazon (*L. p. melanurus*: H_e_ = 0.914; *L. p. aequatorialis*: H_e_ = 0.930). The analysis of possible genetic heterogeneity between these two putative ocelot subspecies in the Colombian Amazon was measured using the F_ST_ statistic, showing an extremely low estimate (F_ST_ = 0.0011), leading to the conclusion that this sample consisted of a single gene pool. In fact, a population assignment analysis using the GENECLASS program showed a low assignment rate for these presumed putative subspecies of Amazonian ocelots, ranging from 0 to 39% of cases. Therefore, it was clarified that these two Amazonian ocelot subspecies constitute a single population. This Colombian Amazonian population and another non-Amazonian Colombian ocelot population, which was classified as the putative subspecies *L. p. pseudopardalis*, showed negative results for bottleneck detection using the Cornuet and Luikart’ method [104]. Conversely, an ocelot population from the Peruvian Amazon showed significant evidence of having recently passed through a bottleneck (in this case, the results were contrary to one preliminary study [46]). The ocelot population in the Colombian Amazon showed no evidence of population expansion when using the Reich and Goldstein g test [105]. Using Nielsen’s method [51], the population size of the ocelot across its entire geographic range was estimated to be between 657,000 and 1,176,000 individuals. These figures fall within the range estimated by Emmons [102] (800,000–3,000,000 ocelots in the Neotropics). **(4)**- An extension of this latter work was that of Ruiz-García et al. [106], which increased the number of ocelots to 294 individuals from 10 different countries, analyzed using 10 nuclear microsatellites. This sample included 142 ocelots of Colombian origin (four from the department of Putumayo, two from the department of Chocó, 12 from the department of Risaralda, 10 from the department of Meta, 16 from the department of Cauca, 14 from the department of Córdoba, eight from the department of Antioquia, two from the department of Cesar, 14 from the department of Amazonas, six from the department of Atlántico, two from the department of Magdalena, four from the department of Guainía, two from the department of Huila, two from the department of Santander, two from the department of Casanare, two from the department of Vichada, six from the department of Vaupés, and 34 ocelots of Colombian origin but without exact geographic origins). Different classification systems for ocelot subspecies that have traditionally been used were adopted. Using the four groupings of Eizirik et al. [100], the population of northwest South America presented the highest genetic diversity (H_e_ = 0.906 ± 0.024, n_A_ = 15.8 ± 4.26), while the one that presented the lowest genetic diversity was the grouping of Central America (H_e_ = 0.815 ± 0.184, n_A_ = 2.9 ± 1.37). Of the 10 microsatellites used, two showed significant genetic heterogeneity in genotype frequencies for the different taxonomic units considered (*FCA45* and *FCA391*). Genetic heterogeneity increased when considering gene frequencies (four microsatellites showed significant differences among the different taxonomic units considered: *FCA45*, *FCA96*, *FCA126*, and *FCA391*). However, the overall F_ST_ statistic (=0.002) was very low, indicating limited genetic heterogeneity among the different putative subspecies or geographic groupings of ocelots, as found by Eizirik et al. [100]. The percentages of correct population assignments were usually low (30–60%), and the number of first-generation migrants showed a high proportion of individuals. In South America, the putative subspecies *aequatorialis*, *pseudopardalis*, *melanura*, and *steinbachi* appear to form a single population. Of all these names, *pseudopardalis* is the oldest. However, this study could not adequately clarify whether *mitis* could be added to this group due to the very small sample size analyzed for this putative subspecies. If it could be added, there would be only one subspecies of ocelot in South America, *L. pardalis mitis* (a conclusion reached by Kitchener et al. [107]). In Central America, another subspecies, distinct from the South American one, *L. pardalis pardalis*, was identified. In fact, a craniometric analysis [108] allowed its author to differentiate two presumed species of ocelot, one for Central America (*Leopardus pardalis*) and another for South America (*Leopardus mitis*). **(5)**- Additionally, Janecka et al. [109,110] analyzed nine ocelots from southern Texas and northern Mexico (Tamaulipas) for the mitochondrial control region, showing that these specimens formed a group clearly distinct from those analyzed by Eizirik et al. [100]. Therefore, it is possible that another subspecies of ocelot, *L. pardalis albiscens*, may be found in North America. Janecka et al. [111] analyzed three ocelot populations [two in Texas, Cameron (n = 52) and Willacy (n = 34), and one in Mexico, Tamaulipas (n = 17)] for 25 microsatellite loci and a 395 bp fragment for the mitochondrial control region. Genetic variation was lowest in the population occurring on Cameron, Texas (nuclear diversity, H_e_ = 0.399 and n_A_ = 2.88; and mtDNA diversity: H_d_ = 0, π = 0, and only one haplotype) and highest in northeastern Mexico, Tamaulipas (nuclear diversity, H_e_ = 0.637 and n_A_ = 4.64; and mtDNA diversity: H_d_ = 0.667, π = 0.0029, and four haplotypes), while intermediate on private lands in Willacy County, Texas (nuclear diversity, H_e_ = 0.553 and n_A_ = 3.72; and mtDNA diversity: H_d_ = 0.252, π = 0.00064, and only two haplotypes). Significant genetic differentiation between the two Texas populations was observed, despite their close proximity (around 30 km). Both populations were also significantly divergent from northeastern Mexico. The absence of any detectable gene flow implies that the human modified landscape of the Lower Rio Grande Valley in southern Texas acts as a strong barrier to ocelot movement, disrupting metapopulation dynamics and contributing to loss of diversity. Janecka et al. [112] used a fragment of the mitochondrial control region (418 bp) and 11 autosomal microsatellite loci to examine historical levels of genetic diversity and infer temporal changes in ocelot populations in Texas between 1853 and 2005. The levels of genetic diversity were higher in historical ocelot populations (museum samples, n = 15) than in extant populations from Texas and Tamaulipas, Mexico (n = 86). For the mitochondrial sequence, the global genetic diversity values for ocelots from Texas and Tamaulipas were H_d_ = 0.254 ± 0.060 and π = 0.00077 ± 0.0002. The historical sample (1853–1956) showed genetic diversity values approximately double the current estimate (H_d_ = 0.543 ± 0.133 and π = 0.00146 ± 0.00043). If we consider only the current Texas ocelot population (H_d_ = 0.163 ± 0.057 and π = 0.00039 ± 0.00014), bearing in mind that the Cameron population currently has a mitochondrial genetic diversity of 0, its diversity is severely reduced. Conversely, the historical Texas population possessed a much higher genetic diversity (H_d_ = 0.673 ± 0.123 and π = 0.00191 ± 0.00049). A similar pattern was observed for the microsatellite loci. The current Cameron and Willacy populations showed H_e_ = 0.389 ± 0.078 and n_A_ = 2.46 ± 0.37 and H_e_ = 0.561 ± 0.042 and n_A_ = 3.18 ± 0.33, respectively. Nevertheless, the historical Texas sample yielded H_e_ = 0.642 ± 0.034 and n_A_ = 3.82 ± 0.33, all these values higher than the extant sample. This supports the argument that low levels of genetic diversity in Texas are related to human-induced population reductions and fragmentation, both of which threaten the remaining ocelots in the USA. However, the levels of genetic diversity, both nuclear and mitochondrial, found in the historical sample are substantially lower than those found in different regions of South America, which is related to the peripatric phenomenon of ocelot populations in Texas and northern Mexico. **(6)-** Rodgers et al. [113] obtained DNA collected noninvasively from feces using capture-recapture modeling. They compared density estimates of ocelots in Barro Colorado (Panama) derived from fecal noninvasive genetic techniques to density estimates from camera trapping in the same population during the same study period. Density estimates from the two techniques were comparable, especially when using spatially explicit capture-recapture models. With four microsatellite loci, 12 unique genotypes and 31 recaptures from 16 latrines were obtained. Six individuals were identified as male, and six as female. All four loci were in Hardy–Weinberg equilibrium, and the number of alleles per locus was 3–6. Based on allele frequencies within the sampled population, probability of individual identity among unrelated individuals P(ID) was 0.00031, and among siblings P(ID)sibs was 0.038. Population density estimated using the program DENSITY was 1.74 ± 0.584 km^2^ from noninvasive genetics and 1.59 ± 0.464 km^2^ from camera trapping. These estimates also represented the highest reported ocelot population density within the species range. **(7)**- Figueiredo et al. [114] analyzed nine microsatellite loci in ocelots inhabiting two Atlantic Forest fragments, Morro do Diabo (n = 14) and Iguaçu Region (n = 18) in southern Brazil. The levels of nuclear genetic diversity were as follows: For Morro do Diabo, H_e_ = 0.709 ± 0.188 and n_A_ = 5.777 ± 2.482, and for Iguaçu Region, H_e_ = 0.704 ± 0.138 and n_A_ = 6.333 ± 2.867. These values can be considered high. The Morro do Diabo ocelot population showed evidence of a genetic bottleneck under two mutational models (Two phase mutation model, TPM: *p* = 0.0371 and SMM: *p* = 0.0488). Estimates of genetic structure (F_ST_ = 0.027; best fit of k = 1 with STRUCTURE) revealed no meaningful differentiation between both populations. Therefore, these results indicate that the ocelot populations sampled in these fragments are still not significantly different genetically, a pattern that strongly contrasts with that previously observed in jaguars in the Brazilian Atlantic Forest. Probably, ocelots have larger effective population size (relative to jaguars) in each fragment, implying a slower effect of drift-induced differentiation, and potentially some remaining permeability of the anthropogenic matrix for ocelots, as opposed to the observed lack of permeability for jaguars. **(8)**- Wultsch et al. [28] carried out an interesting study where they obtained DNA from 1053 droppings of jaguars, pumas and ocelots in Belize, analyzing 14 nuclear microsatellites. Ocelots in Belize showed higher levels of genetic diversity (H_e_ = 0.63 ± 0.03 and n_A_ = 5.11 ± 0.15) than jaguars (H_e_ = 0.57 ± 0.02 and n_A_ = 3.80 ± 0.34) and pumas (H_e_ = 0.57 ± 0.08 and n_A_ = 4.46 ± 1.28). The degree of differentiation among ocelot populations in Belize was somewhat greater than that observed for jaguars, but less than that observed for pumas. AMOVA detected significant, though not excessively large, heterogeneity among sampling sites (F_ST_ = 0.132, *p* < 0.001) and among different regions in Belize (F_ST_ = 0.132, *p* < 0.001). Bayesian clustering analysis in STRUCTURE did not reveal any population subdivision for the ocelots in Belize (k = 1). Bayesian clustering in GENELAND revealed seven genetic clusters, through which individuals were roughly grouped into two main genetic clusters (northern and southern Belize). The northern cluster showed a high degree of admixture, while the southern cluster consisted of several individuals detected at Cockscomb Basin Wildlife Sanctuary that were strongly assigned to this site. Additionally, it was found a significant, but weak signature of isolation-by-distance for the ocelot in Belize (all ocelots, r = 0.134, *p* = 0.020; ocelot males, r = −0.029, *p* = 0.590; ocelot females, *r* = 0.131, p = 0.070), indicating that geographic distance potentially has a small effect on gene flow for this species in that Central American country. **(9)**- Salom-Pérez et al. [115] analyzed 28 ocelots in Costa Rica using 15 nuclear microsatellites. Genetic diversity was high (H_e_ = 0.79 ± 0.08 and n_A_ = 6.87 ± 1.71). An analysis using STRUCTURE and PCoA showed the existence of a single gene pool in that country. However, slight isolation by distance was detected among the Costa Rican ocelots, but overall, no major restrictions on gene flow were found. This species showed higher levels of genetic diversity than the jaguar and puma in that country. The Costa Rican ocelots were genetically distinct from those in Belize (STRUCTURE, k = 2). **(10)**- However, the largest genetic analyses using mitochondrial markers were conducted by Ruiz-García et al. [56] and Ruiz-García et al. (unpublished). In the first study, 309 ocelots were analyzed for four mitochondrial genes (control region, *ATP8*, *16S rRNA*, *ND5*). Of these, 125 ocelots were of Colombian origin. As in most studies conducted with this species, except for the more peripheral populations in northern Mexico and especially Texas, the levels of genetic diversity were high (H_d_ = 0.974 ± 0.005 and π = 0.0306 ± 0.003). The onset of mitochondrial haplotype diversification in the ocelot was estimated to have occurred 2.56–2.09 million years ago. The Mantel test detected a significant relationship between genetic and geographic distances (r = 0.087, *p* < 0.0005), although geographic distance only explained 0.76% of the genetic distances. The global correlogram showed a significant isolation-by-distance structure over a range of approximately 5000 km. Analysis using Monmonier’s algorithm identified 10 main clusters: (1) in the Beni region (Bolivia); (2) in an area of the Loreto department in the Peruvian Amazon; (3) from Caquetá (Colombia) to the northern Ecuadorian and Peruvian Amazon; (4) another area in the Loreto department in the Peruvian Amazon; (5) an area in northern Colombia (Antioquia and Córdoba departments); (6) another from northern Colombia (Bolívar department) to Panama and Costa Rica; (7) another in an area of the Guainía Department (Colombia); (8) another in the Guianas and the island of Trinidad in the Caribbean; (9) another from part of the Beni Department (Bolivia) to the state of Rondônia in the Brazilian Amazon; (10) another local ocelot population in the Loreto Department in the Peruvian Amazon. The mismatch distribution procedure detected a significant population expansion that began approximately 192,000 years ago. The BSP procedure also detected a significant population expansion for this species that began 150,000–100,000 years ago. In the second study, two databases were analyzed. One comprised 340 ocelots sequenced for six mitochondrial genes (139 of which were of Colombian origin), and the other comprised 95 ocelots for their complete mitogenomes (32 of which were of Colombian origin). The first database revealed several clusters with a defined geographic pattern, although the bootstrap values at the nodes of the phylogenetic trees (which determine the robustness of those nodes) were generally low. The main geographic clusters in this analysis were: (1) several overlapping, defined clusters in the Colombian, Ecuadorian, and Peruvian Amazon; (2) a cluster in the western Pacific region of Ecuador; (3) a cluster in northern Panama; (4) several clusters in the Colombian Eastern Llanos; (5) a cluster in southern Peru (Cuzco); (6) a cluster in northern Argentina and Paraguay; (7) a significant cluster in Bolivia; (8) a cluster in the Cundinamarca region (Colombia); (9) several clusters exclusive to the Peruvian Amazon; (10) a cluster in the Cauca Valley (Colombia). (11) a group with specimens from the island of Trinidad along with specimens from eastern Colombia and northern Brazil; (12) a group with ocelots from the department of Córdoba and the department of Chocó (Colombia); (13) a group extending from northern Colombia to the department of Risaralda (Colombia); (14) a group from the central area of Costa Rica; (15) and a group recorded in French Guiana and northern Brazil. With the second database, genetic diversity was determined for the global population (H_d_ = 0.993 ± 0.0004 and π = 0.018 ± 0.00016) and for the Colombian population (H_d_ = 0.991 ± 0.0035 and π = 0.012 + 0.0004). For this database, 15 ocelot pelts seized in Colombia were analyzed, a significant portion of which came from the north-central region and the Pacific coast of that country.

Considering all these genetic studies of the ocelot, Colombian ocelots are well represented. Five of the 15 studies (33%) analyzed included Colombian specimens. In fact, Colombia is the country with the most ocelot samples that have been genetically analyzed. The different genetic and population parameters of ocelots in different regions of their geographic distribution are shown in Table 4.

### 2.5. Margay

The margay (*Leopardus wiedii*) is a small, spotted cat with a wide geographic distribution in tropical America (2.6–4.0 kg in weight and head and body length, excluding the tail, between 48 and 79 cm). Globally, *L. wiedii* is listed as Near Threatened. The current population trend is decreasing, and the population is not severely fragmented.

Morphologically, 10 subspecies of margay have been recognized [82,84,116]:

(1) *Leopardus wiedii amazonicus* (Cabrera, 1917) with its type locality in Tabatinga, Amazonas, Brazil, and with a distribution in a major part of the Amazon River basin, especially the western and central areas of the Amazon.

(2) *Leopardus wiedii boliviae* (Pocock, 1941) with its type locality in Buena Vista, Santa Cruz, Bolivia; Its geographic distribution ranges from Bolivia to northern Argentina.

(3) *Leopardus wiedii glauculus* (Thomas, 1903) with the type locality in Beltrán, Jalisco, Mexico; its distribution extends through Jalisco, Sinaloa, and northern Yucatán in Mexico.

(4) *Leopardus wiedii nicaraguae* (Allen, 1919) with the type locality on the Chinandego volcano, Nicaragua; its distribution is limited to that country.

(5) *Leopardus wiedii oaxacensis* (Nelson and Goldman, 1931) with the type locality in Cerro San Felipe, Oaxaca, Mexico; its distribution is limited to the high mountains of Oaxaca.

(6) *Leopardus wiedii pirrensis* (Goldman, 1914) with the type locality in Cana, Darién, Panama; its distribution is limited to that country.

(7) *Leopardus wiedii salvinia* (Pocock, 1941) with the type locality in Vera Paz, Guatemala; it is distributed throughout Guatemala and probably Belize.

(8) *Leopardus wiedii vigens* (Thomas, 1904) with the type locality in Igarapé-Assu, Pará, Brazil; its distribution is probably in the eastern part of the Brazilian Amazon and the Guianas.

(9) *Leopardus wiedii wiedii* (Schinz, 1821) with the type locality in Morro de Azará, Mucurí River, Bahia, Brazil; its range may extend as far north as the Brazilian state of Espírito Santo.

(10) *Leopardus wiedii yucatanicus* (Nelson and Goldman, 1931) with the type locality in Mérida, Yucatán, Mexico; it is distributed throughout the Yucatán Peninsula and northern Chiapas in Mexico. Wozencraft [96] recognized one more subspecies, following de Oliveira [115]:

(11) *Leopardus wiedii cooperi* (Goldman, 1943) with its type locality in Eagle Pass, Texas (USA) and a distribution extending across Texas (USA) and northeastern Mexico.

Kitchener et al. [107] recognized three subspecies of margay, aligning them with the three molecular groups identified by Eizirik et al. [100], a study we will discuss below. *L. w. wiedii* corresponds to the group found south of the Amazon River in South America, *L. w. vigens* to the group north of the Amazon River in South America, and *L. w. glauculus* to the Central American group. However, Nascimento [108], using biometric studies, found no significant geographic variations in the margay.

The number of molecular studies for the margay is very limited. **(1)**- The first study was by Eizirik et al. [100]. Using 24 specimens from various institutions located in Mexico, Guatemala, Nicaragua, Costa Rica, Panama, French Guiana, Brazil, Bolivia, and Paraguay, and through analysis of the mitochondrial control region (*HVS-I*), they determined exceptionally high levels of genetic diversity for this sample of margays (H_d_ = 0.985 ± 0.018, and π = 0.183 ± 0.092). In fact, this nucleotide diversity was among the highest found for a vertebrate (some sequencing errors in some of the analyzed specimens should be excluded). The authors detected three distinct geographic groupings. The first, in Central America, comprised two subgroups (Mexico-Guatemala and Nicaragua-Costa Rica); The second population is in northeastern South America (northern Brazilian Amazon and French Guiana), and the third south of the Amazon River (eastern and southern Brazil, Bolivia, and Paraguay). Therefore, the Darién Gap and the Amazon River would act as geographical barriers that helped differentiate these three margay populations. At the level of nuclear microsatellites, two studies provided some preliminary results for this species. **(2)**- Ruiz-García [46] analyzed five microsatellites for 14 margays from the Colombian Amazon (n = 12) and the Bolivian department of Santa Cruz de la Sierra (n = 2). Genetic diversity levels were high (H_e_ = 0.846 ± 0.140 and n_A_ = 5.50 ± 1.54). No evidence of bottlenecks was detected. The estimated N_e_ values using two different mutational models were high, ranging from 152,400 to 720,400 individuals. **(3)**- Grisolia et al. [101] analyzed 25 margays from different zoos in Brazil using four nuclear microsatellites. Genetic diversity was also very high (H_e_ = 0.847 ± 0.048 and n_A_ = 11 ± 1.58). **(4)**- However, the study that used a considerably large sample and covered the geographic distribution of this species in 12 different Latin American countries was that of Pinedo and Ruiz-García [117], analyzing 118 specimens for three mitochondrial genes (*ND5*, *16S rRNA*, *ATP8*). Of these 118 specimens, 31 were of Colombian origin (one from the department of Norte de Santander, four from the department of Antioquia, nine from the department of Amazonas, two from the department of Caquetá, four from the department of Chocó, three from the department of Guaviare, two from the department of Magdalena, two from the department of Meta, one from the department of Nariño, one from the department of Valle del Cauca, and two from the department of Vichada). Thirteen clusters were formed in the maximum likelihood tree and 14 clusters in the Bayesian tree, some with a geographic significance and others where margay from different geographic areas appeared intermingled. Among the groupings that had geographical significance, the following appeared: (1) group 4 contained mainly Amazonian specimens (Colombia, Peru, and Brazil) and specimens from the Colombian Eastern Llanos; (2) groups 6 and 9 were of exclusively Amazonian origin (Colombia, Peru, Brazil); (3) groups 7 and 8 of exclusively Bolivian origin; (4) group 11 comprised exclusively of specimens from French Guiana; (5) group 12 was mainly composed of specimens of Amazonian origin (Colombia, Ecuador, Peru) and a couple of specimens from Valle del Cauca and Antioquia; and (6) group 13 was composed exclusively of Central American specimens (Mexico, Guatemala, Costa Rica, and Panama). Many of these groupings do not coincide with the putative morphological subspecies traditionally described. The genetic diversity found for the margay was very high, as previously mentioned (H_d_ = 0.976 ± 0.009 and π = 0.035 ± 0.0032). No spatial genetic structure was found for the margay across its geographic range. Different demographic change statistics, the mismatch distribution procedure and the BSP procedure, showed population expansion for the margay during the Pleistocene. The mismatch distribution procedure determined a population expansion that began between 405,000 and 202,000 years ago, and the BSP procedure detected it between 350,000 and 300,000 years ago, with a sharp increase in expansion 150,000 years ago. **(5)**- Ruiz-García et al. [56] analyzed the same set of samples as before but increased the analysis to include one more mitochondrial marker (control region). The results for genetic diversity, spatial structure, and population expansions were practically the same as in the previous study. Two novel results in Ruiz-García et al. [56] for the margay were, firstly, the initial period of diversification of mitochondrial haplotypes estimated between 2.58 and 1.91 million years ago (beginning of the Pleistocene), and secondly, the determination of geographic areas that contained specimens with certain genetic differences using the Monmonier algorithm: (1) Bolivian departments of Cochabamba and Chuquisaca; (2) from Tena (Ecuador), through the department of Nariño (Colombia) to southern Costa Rica; (3) the department of Loreto in the Peruvian Amazon; (4) from the northern Pacific coast of Colombia (departments of Chocó and Antioquia) to Chitré (Panama); (5) another grouping in the department of Loreto in the Peruvian Amazon; (6) the Eastern Llanos and Colombian Amazon (from the department of Vichada to the department of Amazonas); (7) from Cobán (Guatemala) to San Luis Potosí in northern Mexico; and (8) another grouping in the Peruvian Amazon, in this case, in the department of Ucayali. Many of these small geographic groupings did not necessarily coincide with the proposed morphological subspecies.

Therefore, there are not many published molecular studies on the margay, but the two most extensive [56,117] include a good representation of Colombian specimens, as margays of Colombian origin were present in three of the five articles analyzed for the margay (60%). Table 5 compares the genetic and population parameters of this species analyzed in different regions of the Neotropics.

### 2.6. Tigrina or Oncilla

The tigrina or oncilla (traditionally *Leopardus tigrinus*) is the smallest feline found in Colombia (weighing 1.5–3.0 kg and measuring 45–65 cm in head and body length, excluding the tail). Globally, *L. tigrinus* is listed as Vulnerable under criteria A2c. The current population trend is decreasing, and the population is severely fragmented.

The nomenclature of this feline species has been confusing since its origins and became even more so in the early decades of the 20th century. Schreber [118,119] first used the scientific name *Felis tigrina* and illustrated this species with a plate named “Le Margay” [120]. It was based on a specimen from Cayenne, French Guiana. Thus, biologists have long been confused about what a tigrina is and what a margay is. Gray [121] described a supposed new species, *Felis pardinoides*, with “India” as its type locality, but later changed the type locality to Bogota, Colombia [122]. In the same period, *Felis guttula* was described, and southern Brazil was listed as its type locality [123]. Additional tigrina taxa were described at the beginning of the 20th century, including *Felis pardinoides oncilla* (Costa Rica), *Felis pardinoides andina* (Ecuador), *Felis carrikeri* (Costa Rica), *Felis pardinoides emerita* (Venezuela), and *Felis emiliae* (Ceara State, Brazil). Allen [124] used the genus *Margay* for the tigrina and defined two additional tigrina taxa: *Margay tigrina elenae* (department of Antioquia, Colombia) and *Margay caucensis* (department of Cauca, Colombia). The traditional taxonomy that was used until recently was proposed by Cabrera, [82,125] and followed by Wozencraft [98]. It comprised four subspecies:

(1) *Leopardus tigrinus oncilla* (Thomas, 1903; including *F. p. oncilla* and *F. carrikeri*); This taxon inhabits Panama, Costa Rica as far north as Nicaragua.

(2) *Leopardus tigrinus pardinoides* (Gray, 1867; including *F. pardinoides*, *F. p. andina*, *F. p. emerita*, *M. t. elenae*, and *M. caucensis*); This taxon inhabits the Andean region including western Venezuela, Colombia, Ecuador, Peru, and possibly Bolivia, and northwestern Argentina.

(3) *Leopardus tigrinus tigrinus* (Schreber, 1775; including *F. tigrina* and *F. emiliae*); This taxon lives in eastern Venezuela, the Guianas, and northeastern Brazil.

(4) *Leopardus tigrinus guttulus* (Hensel, 1872; including *F. guttula*), which would live in the central and southern areas of Brazil, Paraguay and Argentina (province of Misiones). However, the last subspecies was claimed to be a new species (*Leopardus guttulus*) based on genetic data, as we will discuss shortly.

Pelage characteristics and morphometrics from a large collection of *L. tigrinus* support the existence of three morphogroups [126]. Morphogroup I contains specimens from Central America, as well as specimens from northern, northwestern, and western South America (Costa Rica, Colombia, Venezuela, Ecuador, Peru, Guyana, Suriname, and northwestern Argentina). The color of these tigrinas is brown and ranges from orangish brown to yellowish-brown. These specimens have a greyish-brown underfur, a white or light gray venter, and medium-sized rosettes that form oblique bands. The bands are arranged in a scapular-inguinal direction on the sides of the body. This taxon is *L. tigrinus* in “senso stricto”. Morphogroup II contains specimens from the northeastern and central Brazil area. Their color ranges from a light yellowish-brown to pale yellow or pale greyish buff. They have small rosettes that rarely form oblique bands. The rosettes have thin and discontinuous black rims. This morphogroup was considered a new species, *Leopardus emiliae* [126]. Morphogroup III contains specimens from southern Brazil, Paraguay, and northeastern Argentina and corresponds to *L. guttulus*. They have a dark yellowish-brown ground color, which is lighter on the sides of the body. They also have a white or light gray venter and small rosettes on the sides of the body.

**Figure 8:**
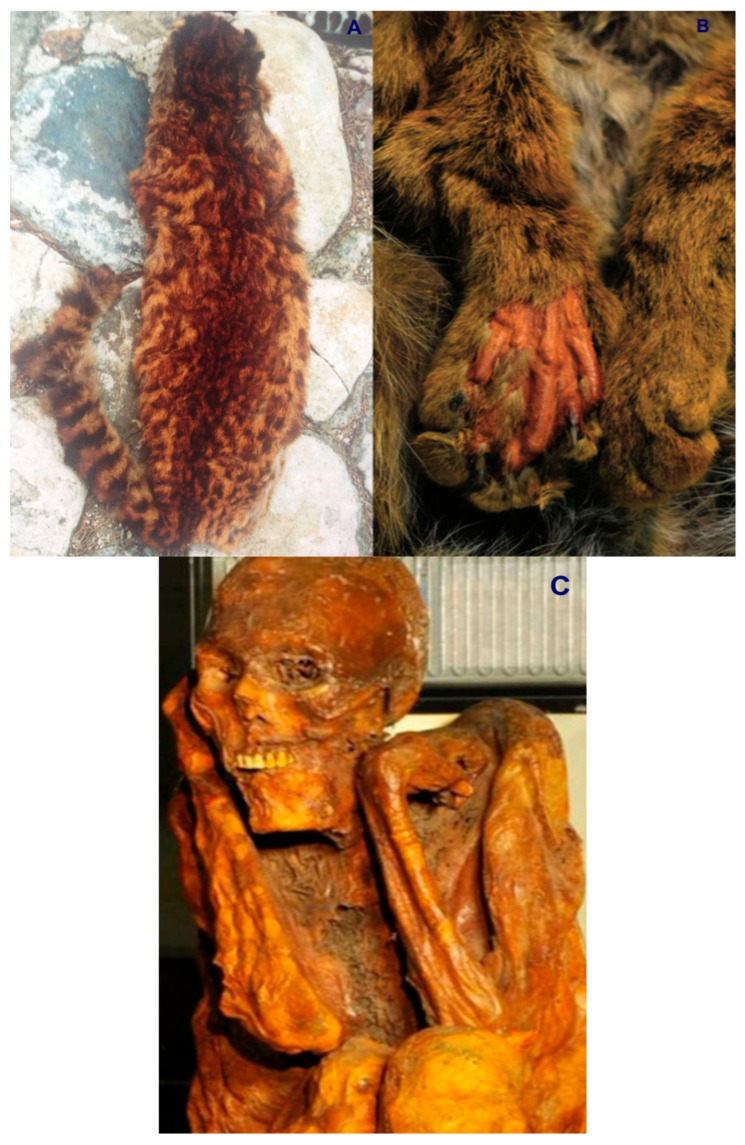
Nariño cat (tentatively *Leopardus narinensis*). (**A**) The holotype of the Nariño cat (specimen 5857 from the von Humboldt Institute in Villa de Leyva, Boyacá, Colombia). (**B**) Mummified leg of the Nariño cat. (**C**) A human mummy discovered at 3000 m above sea level in the Peruvian Andes, at the source of the Ucayali River, deposited in the Vatican and repatriated to Peru in recent years. The red coloration of the Nariño cat’s paw is practically identical to the red epithelial and muscle tissue of certain human mummies.

Various molecular studies have attempted to clarify the taxonomy and systematics of tigrinas, but the situation is complex and requires further studies to satisfactorily resolve the issue. **(1)**- Johnson et al. [127] detected strong genetic differentiation between four specimens from Costa Rica and 28 specimens of Brazilian origin (southeastern Brazil) by studying mitochondrial sequences (*ATP8*, *16S rRNA*, and *ND5*), with a genetic divergence of almost 5%, which, for the first time, suggested species-level distinction between the two populations. Likewise, the authors detected evidence of natural hybridization between *L. tigrinus* and the Pampas cat, *Leopardus colocola*. The hybridization occurred in areas of central Brazil where the ranges of the species overlapped. They detected nine *L. tigrinus* specimens with mitochondria from *L. colocola*. Of these nine individuals, three were males with identical ZFY haplotypes of a tigrina from southern Brazil. This supports the conclusion that these animals were hybrids of *L. tigrinus* males and *L. colocola* females. However, Trigo et al. [128,129,130] were the first that unambiguously showed the existence of at least one fully differentiated species within the traditional tigrina. **(2)**- In the first work, using sequences of the mitochondrial genes *ND5*, *ATP8* and control region and nine microsatellites, these authors detected and characterized a hybrid zone between the southern tigrina (*L. guttulus*) (n = 57) and Geoffroy’s cat (*Leopardus geoffroyi*) (n = 41) in southern Brazil. Genetic diversity levels for both species were high (*L. guttulus*: H_d_ = 0.9266 ± 0.0247 and π = 0.0039 ± 0.00227; *L. geoffroyi*: H_d_ = 0.8990 ± 0.0341 and π = 0.0062 ± 0.0034). Eight specimens of *L. geoffroyi* carried mitochondrial DNA from *L. guttulus*, and one Pampas cat (*L. colocola*) also carried mitochondrial DNA from *L. guttulus*. In turn, six specimens of *L. guttulus* carried mitochondrial DNA from *L. geoffroyi*, and five tigrinas carried mtDNA from *L. colocola*. For microsatellite markers, genetic diversities were also high (*L. guttulus*: H_e_ = 0.716 ± 0.141 and n_A_ = 8.89 ± 4.507; *L. geoffroyi*: H_e_ = 0.714 ± 0.167 and n_A_ = 8.22 ± 6.087). **(3)**- In the second work, the authors show strong evidence of ancient hybridization and introgression between the Pampas cat (*L. colocola*) and northeastern populations of tigrina (*L. tigrinus*, or maybe *L. emiliae*), leading to remarkable cytonuclear discordance in the latter. In contrast, southern tigrina populations show recent and continuing hybridization with Geoffroy’s cat (*L. geoffroyi*), leading to extreme levels of interspecific admixture at their contact zone. To do this, they used four datasets. Likewise, the authors demonstrated that two apparently continuous Brazilian tigrina populations show no evidence of ongoing gene flow between them, supporting the existence of two species of tigrina in Brazil, *L. tigrinus* in the northeast and *L. guttulus* in the south, which were separated around 0.5–0.8 million years ago. **(4)**- The third study expanded the analysis of the hybridization among *L. guttulus*, *L. geoffroyi* and *L. colocola*. For the first time, authors provided strong evidence of ancient hybridization and introgression between *L. colocola* and populations of tigrina (*L. t. tigrinus*) in northeastern Brazil. In contrast, southern Brazilian tigrina populations engaged in recent and continuing hybridizations with *L. geoffroyi*. They concluded that these results indicated that the admixture between these species is quite concentrated in the areas surrounding their contact zone, suggesting either that the hybridization process is extremely recent and/or that there is some selective restriction on the geographical spread of admixed descendants outside this contact zone in southern Brazil. Additionally, they concluded that both feline species (*L. guttulus* and *L. geoffroyi*) evolved in allopatry around one million years ago. Furthermore, the hybridization process occurred very recently because they detected population expansions with different procedures around 76,000 years ago for both feline species. This population expansion was stronger in tigrina than in Geoffroyi’s cat. However, the authors did not discard a much more recent hybridization influenced by human activities. Rampant anthropogenic habitat alteration has occurred in some areas of southern Brazil for over two centuries. It is conceivable that these populations were not indirect contact prior to human disturbance. Depending on the intensity of interspecies breeding per generation, the authors claimed that it is possible that two centuries of hybridization could lead to the observed pattern of admixture. **(5)**- The first molecular study focused specifically on the Andean tigrina was carried out by Ruiz-García et al. [131]. In that study, two databases were analyzed. The first consisted of 41 tigrinas (including 23 animals of Colombian origin) sequenced for two mitochondrial genes (*ATP8* and *16S rRNA*). The second consisted of 18 tigrinas (seven of Colombian origin) sequenced for their complete mitogenomes. The most striking results of that first molecular study with the Andean tigrina were (1) the relationship between the Central American tigrinas and several tigrinas from the trans-Andean (Pacific) region of Colombia and Ecuador, (2) the existence of several independent mitochondrial lineages [one relating a Peruvian specimen and two Colombian specimens (departments of Cundinamarca and Tolima), and another lineage comprising Colombian individuals from the departments of Quindío, Boyacá, and Huila and one individual from the province of Cotopaxi in Ecuador], (3) a significant proportion of the Colombian tigrinas exhibited mitochondrial haplotypes similar to those of the margay and the ocelot. This was also found in some tigrinas from Venezuela, Ecuador, Bolivia, and northwestern Argentina. In some cases, these could be ocelots and margays that, living at high altitudes, exhibited phenotypes with certain phenotypic convergences to Andean tigrinas, but in other cases, they have been shown to be tigrinas with ancient genetic introgression from ocelot and margay ancestors. (4) Perhaps the most striking finding was the detection of a specimen with fur characteristics clearly differentiated from other tigrinas (specimen 5857 from the von Humboldt Institute in Villa de Leyva, Boyacá, Colombia), which formed a unique phylogenetic branch separate from the other tigrinas groups. It was not formally named in that study. However, work continued with this individual until its complete mitogenome was obtained, in addition to the analysis of 30 tigrinas for the mt*ND5* gene and the application of six nuclear microsatellites. **(6)**- Ruiz-García et al. [132] published comparative results between this unusual-looking pelt from the Galeras volcano (Nariño department, Colombia) and the other tigrinas studied, suggesting the possibility that it represented a new species within the genus *Leopardus*, *Leopardus narinensis*. Results using the mt*ND5* gene placed the Nariño cat (as it came to be called) as a sister taxon to *L. colocola*, while mitogenomic data and microsatellites placed it as the sister group to the Central American and trans-Andean tigrinas of Colombia and Ecuador, plus (*L. geoffroyi* + *Leopardus guigna*). The news received considerable coverage in the Colombian press. However, several researchers criticized that the Nariño cat was, in fact, a distinct species within the genus *Leopardus*. The initial criticisms (and criticisms are very important in science) were not very orthodox because they did not follow good scientific practice. Those who made them did not use the appropriate scientific channels but instead relied on social media and some journalists eager for attention to criticize the work of Ruiz-García et al. [132]. Some of these criticisms revealed that those who made them used these mass media outlets to introduce their own poor scientific practices. For example, they accused the Ruiz-García’s team of not uploading all the sequences generated in their work to GenBank, but they themselves never uploaded to GenBank the sequences they supposedly obtained from the Nariño cat, which, according to them, showed that it was not genetically different from the “usual” Andean tigrinas. Subsequently, Astorquiza et al. [133], and more recently Marín-Puerta et al. [134], criticized the existence of *L. narinensis*. **(7)-** However, the recent publication by Ruiz-García et al. [135] revealed several extremely important findings for understanding the complex systematic status of tigrinas in the Andean region. On the one hand, it showed that it is unclear why different temporal analyses of the same skin, conducted in 2001, 2007, 2017, and 2023 using the same genetic markers, yielded different results. The 2001 and 2007 analyses (carried out in Bogotá and Canada, respectively) produced the same results, indicating that the Nariño cat could be a new taxon within the genus *Leopardus*. However, the 2017 analysis placed it closer to *L. colocola*, and the 2023 analysis (after Astorquiza et al. [133], also used the same skin for their research) placed it phylogenetically closer to other Andean tigrina specimens. We hypothesized that the 2017 and 2023 samples may have been contaminated with DNA from other *Leopardus* specimens, specifically from the Pampas cat in 2017 (at that time we were working with samples of this species in the laboratory alongside the Nariño cat sample) and from the Andean tigrina in 2023 (since we also began working in the same laboratory that year with samples obtained from new Andean tigrinas). If this were the case, the older samples, which yielded identical results, would identify the Nariño cat as a new taxon. On the other hand, Ruiz-García et al., [135] showed that, although the Nariño cat did not constitute a new species (despite its phenotypic appearance clearly differentiated from the “usual” Andean tigrina), the arguments of Astorquiza et al., [133] and Marín-Puerta et al., [134], by which the Nariño cat would not constitute a new taxon, are full of errors and biased interpretations: (1) they compared the sequences they obtained from the Nariño cat (without knowing if they were contaminated with DNA from the Andean tigrina or not) with only two pampas cats and two Andean tigrinas [133] and with two Andean tigrinas (the same ones), a Central American tigrina, a *L. guttulus*, *a L. t. emiliae* and a *L. colocola* [133]. Because the taxa of the genus *Leopardus* have undergone rapid and recent speciative radiation, along with the historical introgression and recent hybridization that has commonly occurred among the different taxa of this lineage, it is very important to compare many specimens of all the taxa recognized to date within the genus *Leopardus*. The comparisons by those authors were made with very few specimens. Clearly, the three northern Andean and Central American tigrinas used by Astorquiza et al. [133], Lescroart et al. [136] and Marín-Puerta et al., [134] represented the majority group of Andean tigrinas, but they totally underestimated the genetic diversity of minority tigrina groups that were found by Ruiz-García et al., [135] (see below) that went completely unnoticed in their sampling. Previously, Ramírez et al. [137] carried out a population genetic and phylogenetic analysis of *Leopardus* based on genome-wide SNP data. They found three molecularly distinct groups of tigrinas. On the one hand, five tigrinas from southern Brazil (*L. guttulus*) formed a compact cluster, with two tigrinas specimens from northeastern Brazil (which they did not assign a specific nomenclature to) as a sister group (but highly differentiated from the first). Both clusters behave as the sister group to *L. guigna* and *L. geoffroyi*. Outside of this clade formed by the two Brazilian tigrina forms, plus *L. guigna* and *L. geoffroyi*, a Central American tigrina appears. More recently, Lescroart et al. [136] have further explored the relationships between different *Leopardus* species. It is interesting to note some of the results obtained in this last work. In their phylogenomic analysis, they showed that two tigrinas from eastern Brazil (potentially *L. tigrinus emiliae* or *L. emiliae*) differ from the tigrina of southern Brazil (*L. guttulus*), although both are sister taxa that, in turn, are more closely related to Geoffroy’s cat (*L. geoffroyi*) and the kodkod (*L. guigna*) than to the two tigrinas from Colombia and the tigrina from Costa Rica, which are also sister taxa between them. Conversely, when using the same specimens but for their complete mitogenomes, the relationship between the two Colombian tigrinas and the Central American tigrina was preserved, but the specimen from southern Brazil (*L. guttulus*) was added as a sister group to these three tigrinas. In this case, all the tigrinas showed a more pronounced phylogenetic relationship with each other, while the clade formed by *L. geoffroyi* and *L. guigna* behaved as a clade more external to them. The two tigrinas from eastern Brazil, having introgressed mitochondrial DNA from Pampas cat, were associated with them. That is, nuclear and mitochondrial phylogenies revealed different relationships among the various tigrina taxa. (2) Astorquiza et al. [133] and Marín-Puerta et al. [134] analyzed only 845 bp compared to the 16,756 bp used by Ruiz-García et al. [132,135]. In fact, of the markers used by the former authors, the one with the greatest discriminatory power is mt*ND5*, but they only sequenced 145 bp of that gene, whereas the other authors usually use a minimum of 315 bp, if not the entire 1800 bp that make up that gene; (3) Astorquiza et al. [133] and Marín-Puerta et al. [134] stated that the Nariño cat is 100% genetically identical to the two Andean tigrinas they used in their analyses. However, the supplementary tables of Astorquiza et al. [133] are riddled with easily identifiable calculation errors that show the Nariño cat is not 100% genetically identical to those two tigrinas (one from the department of Caldas and the other from the department of Antioquia). Although the phylogenetic tree of Marín-Puerta et al. [134] showed that the Nariño cat is on a different branch than the two aforementioned Andean tigrinas, they yet continued to claim that they are 100% identical. It is obvious that if the three specimens were 100% identical, they would form an unresolved tritomy in the phylogenetic tree, and the Nariño cat would not be on a different branch. (4) The argument of Astorquiza et al. [133] and Marín-Puerta et al. [134] that the Nariño cat is classified within morphotype I [126] is insufficient to demonstrate that it could not be a taxon distinct from the “traditional” Andean tigrina. A margay could also be classified within morphotype I of the cited authors, yet it is not the same species as the Andean tigrina. Another unusual argument of Marín-Puerta et al. [134] is that the Nariño cat’s fur has a strange appearance because it has been smoked. It is true that one of the Nariño cat’s paws has a striking red patch. They compare the uniqueness of this paw with several smoked skulls, but it is easily verified that they are entirely different shades of red (blackish red in the case of the smoked skull). However, if those authors had simply compared the red coloration of the Nariño cat’s paw with the red epithelial and muscle tissue of certain human mummies (for example, the mummy of Juanita from the Ampato volcano in Arequipa, Peru and the human mummies discovered at 3000 m above sea level in the Peruvian Andes, at the source of the Ucayali River, deposited in the Vatican and repatriated to Peru in recent years, see Figure 8), they could easily figure it out that the Nariño cat’s paw is mummified, a process typical of high-altitude environments, especially those that are dry and cold. It would be interesting for an archaeologist specializing in Andean mummies to study the Nariño cat’s skin. Finally, Ruiz-García et al. [135] showed that not only is the Nariño cat an unusual tigrina in phylogenetic trees with the mt*ND5* gene and the whole mitogenomes, but that other specimens “a priori” tigrinas occupy unusual positions in different branches of the phylogenetic trees. For the mt*ND5* gene, up to six branches or groupings (excluding the Nariño cat) were detected outside the clade containing the “usual” Andean tigrinas, which were designated G1 [(1) a branch of tigrina specimens introgressed by ocelot and margay, (2) two unusual Ecuadorian tigrinas, (3) two unusual Colombian tigrinas, and (4) a group of tigrinas designated G2, genetically highly differentiated from the G1 group]. For the mitogenomes, up to eight branches or groups (excluding the Nariño cat) were detected with unusual positions in the phylogenetic trees [(1) the introgressed tigrina specimens by ocelot and margay, (2) four unusual Colombian tigrinas, (3) two unusual Ecuadorian tigrinas, and (4) the G2 group]. To further complicate matters, de Oliveira et al. [138] have begun using the binomen *Leopardus pardinoides* for the Andean tigrina, in the same way that *L. tigrinus guttulus* was elevated to *L. guttulus*. However, no genetic research, like that conducted with the southern Brazilian tigrina, has been carried out to determine whether this is indeed the correct scientific name for the Andean tigrina if only one molecular lineage of this taxon existed. However, it is quite possible that more than one molecular lineage of tigrinas exists in the Northern Andes. To determine this, the holotype would need to be sequenced, or a neotype created from a modern tigrina captured near Bogotá (which is apparently where the holotype of *pardinoides* originated). In fact, with reference to Tigrina G1, here is a brief commentary. Within this cluster, we detected two sub-clusters, one composed of specimens mainly sampled at the Eastern Colombian Andean cordillera and another composed of specimens sampled in the Central and Western Colombian Andean cordilleras as well as in the Ecuadorian Andean cordilleras. The first sub-cluster should be named *Leopardus tigrinus pardinoides* “sensu stricto” or *Leopardus pardinoides pardinoides* [138], because the holotype of *pardinoides* is thought to be from the Eastern Andean Colombian cordillera in Bogota [122]. The second sub cluster should be named *Leopardus tigrinus oncilla* or *Leopardus pardinoides oncilla* [138] (the holotype with type locality: Volcán de Irazu, Costa Rica [139]). Within this sub-cluster were the two Central American tigrinas analyzed by Ruiz-García et al., [135] as well as the two Colombian tigrinas analyzed by Astorquiza et al. [133] and Lescroart et al. [136] and other tigrinas from Colombia (Western and Central Cordilleras) and Ecuador. With this result, the proposal by Marín Puerta et al. [134] that the two Colombian Andean tigrinas analyzed by Astorquiza et al. [133] (which do not come from the Eastern Andean Colombian Cordillera) could be classified as *Leopardus pardinoides pardinoides* is unfeasible. Both the Central American and the Andean tigrina analyzed by Astorquiza et al. [133] and Marín-Puerta [134] would be classified as *L. pardinoides oncilla*, and the scientific name *L. pardinoides pardinoides* would only be intended for the tigrinas from the Eastern Andean Colombian Cordillera.

For the Andean tigrina, the only accurate estimates of genetic diversity are those derived from Ruiz-García et al. [135]. The genetic diversity estimates obtained for the mt*ND5* gene for tigrinas G1 and G2 were, respectively, the following: H_d_ = 0.958 ± 0.036 and π = 0.0188 ± 0.0051 and H_d_ = 1.000 ± 0.045 and π = 0.0583 ± 0.0126. For the complete mitogenomes, these genetic diversity estimates for tigrinas G1 and G2 were, respectively: H_d_ = 0.949 ± 0.005 and π = 0.0101 ± 0.00018 and H_d_ = 1.000 ± 0.005 and π = 0.0594 ± 0.0006. These results show very high levels of genetic diversity, both within group G1 and, especially, within group G2. Well-differentiated genetic lineages (or even species) may exist, especially within G2, and only with intensive sampling will the systematics of Andean tigrinas be definitively clarified.

At this time, we do not know how many distinct forms of Andean tigrinas exist in the Colombian, Ecuadorian, and Peruvian Andes, nor do we know precisely which scientific nomenclature should be used. This greatly hinders the biological conservation of these small, spotted felines, which are also not common in the Northern Andes. Table 6 shows a comparative analysis of some genetic and population parameters for the two groups of tigrinas that have been analyzed in greater depth (the southern Brazilian tigrina, *L. guttulus*, and the tigrinas of the Northern Andes). It is a priority to continue analyzing these latter populations in depth, in addition to the tigrina populations of northeastern Brazil, Guianas, Venezuela and Central America (Costa Rica and Panama).

### 2.7. Pampas Cat

The situation is complex for the Pampas cat, also known as the colocolo cat (here we will refer to it as *Leopardus colocola*, although since the study by Nascimento et al. [140], the scientific name *Leopardus garleppi* has begun to be used for the most northern taxon of the Pampas cat). It is a small feline (weighing 2–3.7 kg and measuring between 52 and 70 cm in head and body length, excluding the tail). Globally, *L. colocola* is listed as Near Threatened. The current population trend is decreasing, and it is unknown whether the population is severely fragmented.

Traditionally, many different morphotypes (up to eight) of this taxon, distributed across Ecuador, Peru, Bolivia, Chile, Argentina, Paraguay, Brazil, and Uruguay, were classified as a single species, *Felis colocola*, [141] (see the discussion by Kitchener et al. [106], on why *colocola* should be used instead of *colocolo*).

García-Perea [142] conducted a major review of skulls and skins from 86 specimens and concluded that the pampas cat included three distinct species and 10 subspecies:

(1) *Leopradus colocola* (Molina, 1782). Type locality: “Boschi del Chili”, restricted to the province of Valparaiso by Osgood [143]. This species would contain two subspecies: *L. c. colocolo* (Molina, 1782) in the central Chilean provinces, and *L. c. wolffsohni* (García-Perea, 1994) living in the highlands of Tarapacá province (northern Chile), western slope of the Andes.

(2) *Leopardus pajeros* (Desmarest, 1816). Type locality: pampa from Buenos Aires, between 35 and 36°, with a wide distribution from the highlands on the eastern slope of the Andes in Ecuador, Peru, Bolivia, and northwestern Argentina and lowlands of northwestern, central, and southern Argentina, and Chilean Patagonia. This species should contain the following subspecies *L. p. pajeros* (Desmarest, 1816; distributed by La Pampa province in central Argentina), *L. p. budini* (Pocock, 1941; distributed by the mountains of northwestern Argentina, eastern side of Andes and with the type locality in Mount Sola in the Salta province in Argentina), *L. p. crespoi* (Cabrera, 1957; with the type locality in Aguaray, Salta province), *L. p. cruscinus* (Thomas, 1901; distributed in southern half of Argentina and Chilean Patagonia with type locality in Santa Cruz, Argentina), *L. p. garleppi* (Matschie, 1912; distributed by the highland steppes of Peruvian Andes, eastern side with the type locality in Cuzco, and Apurimac in Peru), *L. p. steinbachi* (Pocock, 1941; distributed by the highland steppes of Bolivian Andes, eastern slope with type locality in Tiraque, Cochabamba, Bolivia), and *L. p. thomasi* (Lönnberg, 1913; distributed by the highland steppes of Ecuadorian Andes, eastern side with type locality near Quito, Ecuador).

(3) *Leopardus braccatus* (Cope, 1889). Type locality the province of Rio Grande do Sul, or in Matto Grosso, restricted by Allen [91] to Chapada, Mato Grosso, Brazil. This species should contain two subspecies, *L. b. braccatus* (Cope, 1889; distributed in the southwestern, and central Brazil (Mato Grosso and Mato Grosso do Sul) and in Paraguay with type locality in Chapada, Matto Grosso in Beazil), and *L. b. munoai* (Ximenez, 1961; distributed in southern Brazil (Rio Grande do Sul) and in Uruguay with type locality in Arroyo Perdido, Doriano department, Uruguay).

Subsequently, Kitchener et al. [107] proposed the following classification scheme, with a single species of Pampas cat:

(1) *L. colocola colocola* (geographical distribution as in the previous case).

(2) *L. colocola wolffsohni* (geographical distribution as in the previous case).

(3) *L. colocola pajeros* (including *cruscinus*; geographical distribution by northcentral central and southern Argentina).

(4) *L. colocola budini* (including *steinbachi* and *crespoi*; geographical distribution in northwestern Argentina and Bolivian Andes and eastern to the Andes).

(5) *L. colocola garleppi* (including *thomasi*; geographical distribution possibly southern Colombia, Ecuador, and Peruvian Andes). However, García-Perea [142] claimed that *thomasi* is conspicuously smaller than *garleppi* and their ectotympanic chamber is smaller than in *garleppi*.

(6) *L. colocola braccatus* (geographical distribution as in the previous case).

(7) *L. colocola munoai* (basically distributed in Uruguay).

**(1)**- The first molecular study conducted on the Pampas cat, which included 22 samples from Brazil, Uruguay, northern and central Chile, Bolivia, and Argentina, analyzing three mitochondrial genes (*ND5*, *16S rRNA*, *ATP8*) [127], concluded that it was a single species. Although that study identified three genetically distinct, geographically structured groups (Argentina and central Chile; Bolivia and northern Chile; Uruguay and southern Brazil) and that the common ancestor of these three populations diverged 1.7 million years ago, the levels of intraspecific genetic variation were relatively low (only 2.3%, lower, for example, than what was found in the Geoffroy’s cat, *L. geoffroyi*, or the tigrina, *L. tigrinus*, in that same study). **(2)**- Similarly, Cossíos et al. [144] conducted a study with 199 DNA samples of *L. colocola* from 19 different populations (eight Peruvian, five Bolivian, and six Argentinian populations) using mitochondrial gene sequences (*HVS-I* control region, *ND5*, and *ATP8*) and five nuclear microsatellites. The phylogenetic analysis determined the existence of four well-differentiated genetic groups: (1) a clade A made up of specimens of Peruvian and northern Bolivian origin, corresponding to *L. colocola garleppi* (sensu Kitchener et al. [107]); (2) a clade B made up of specimens from northern Chile, *L. colocola wolffshoni*, but also including, in this clade, some specimens from southern Bolivia-northern Argentina, *L. colocola budini*, and some specimens from Argentina, *L. colocola pajeros*; (3) a clade C typical of specimens from central Bolivia, *L. colocola steinbachi*, and southern Bolivia and northern Argentina, *L. colocola budini*, as well as some specimens from central Argentina, *L. colocola pajeros*, and (4) a clade D that was typically found in central Argentina, *L. colocola pajeros*, and exclusively in southern Argentina, *L. colocola cruscinus*. As can be seen, in southern Bolivia and northern Argentina, four of these typical haplogroups converge with different taxa: *L. colocola steinbachi*, *L. colocola wolffshoni*, *L. colocola budini*, and *L. colocola pajeros-cruscinus*. An analysis using STRUCTURE and AMOVA, with both mitochondrial sequences and microsatellites, detected three different clusters: (1) the first corresponded to all Peruvian populations and two populations from northern Bolivia (primarily *L. c. garleppi*); (2) the second to two populations from south-central Bolivia (Potosí and Tarija) and one from northern Argentina (Jujuy) (where *L. colocola steinbachi* and *L. colocola budini* would converge); and (3) the third would include five Argentinian populations (*L. colocola budini*, *L. colocola pajeros*, *L. colocola cruscinus*). The only difference between mitochondrial genes and microsatellites would be the position of population 12 (Cochabamba, Bolivia). Mitochondrial genes placed it in cluster 2, while microsatellites placed it in cluster 3. Global genetic diversity was very high for both mitochondrial DNA and microsatellites (H_d_ = 0.94 ± 0.034, π = 0.0609 ± 0.0173, H_e_ = 0.836 ± 0.043, and n_A_ = 14.80 ± 3.544, respectively). The temporal divergence of the four clades is thought to have originated during the pre-Pastonian glacial period of the Pleistocene (1.3–0.8 million years ago), and the proliferation of haplotypes within the four clades is thought to have occurred between the Aftonian interglacial period and the end of the Kansas glacial period (0.62–0.30 million years ago), all within the Pleistocene. **(3)**- Ruiz-García et al. [145] expanded the previous study, with 235 pampas cat specimens for mitochondrial sequences of the *HVS-1* control region and for five nuclear microsatellites. The levels of genetic diversity were high for both types of molecular markers (mtDNA: H_d_ = 0.932 ± 0.007, and π = 0.0513 ± 0.0016; microsatellites: H_e_ = 0.741 ± 0.064 and n_A_ = 7.6 ± 3.48). The geographic area exhibiting the greatest genetic diversity corresponded to that of *L. colocola budini* (south-central Bolivia and northern Argentina), confirming the findings of the previous study (mtDNA: H_d_ = 0.903 ± 0.023, and π = 0.0525 ± 0.0011; microsatellites: H_e_ = 0.828 ± 0.049 and n_A_ = 12.20 ± 2.65). This can be interpreted as this being the original expansion area of the Pampas cat or a geographic area where several different gene pools of this feline species converge. Genetic heterogeneity among the five Pampas cat subspecies considered in this study (*garleppi*, *steinbachi*, *budini*, *pajeros*, and *cruscinus*) was significant (F_ST_ = 0.132 ± 0.023, *p* < 0.001). Only the *pajeros*-*cruscinus* pair showed no evidence of significant differentiation. Geographic assignment in this study, when considering all 14 analyzed populations, showed moderately low percentages (ranging from 49.2% to 55.3%). However, when the same test was performed considering the subspecies, the percentage of correct assignment increased considerably (69.8–82.4%). For the geographic range considered, microsatellites showed significant evidence of population expansion for this species. The test by Zhivotovsky et al. [146] showed evidence of population expansion for the sample as a whole (S_k_ = 0.721, t = 2.999, 4 df, *p* < 0.05) and particularly for the three subspecies distributed furthest south: *budini* (S_k_ = 0.611, t = 2.858, 4 df, *p* < 0.05), *pajeros* (S_k_ = 0.429, t = 2.754, 4 df, *p* < 0.05), and *cruscinus* (S_k_ = 0.616, t = 2.974, 4 df, *p* < 0.05). Therefore, the three Argentinean subspecies showed clearer evidence of population expansion. However, unlike microsatellites, mtDNA did not provide consistent evidence of population expansion. The subspecies *garleppi* showed some evidence of population expansion (for Fu and Li D* and Fu and Li F* tests), while for *steinbachi*, some evidence of bottlenecks was discovered (for Fu’s Fs and rg tests). *Budini* and *pajeros-cruscinus* did not show any evidence of demographic changes. The historical effective number of pampas cats in the considered geographic area was estimated, ranging from 82,800 to 328,000 individuals, depending on the different procedures used. A strong and significant spatial structure for the pampas cat was also evident using the Mantel test, isolation models by distance (IBD program), and spatial autocorrelation. The Mantel test significantly showed that 35% of the genetic distances between pairs of Pampas cat populations were due to geographic distances (r = 0.588; approximate Mantel t-test, t = 4.926, *p* = 0.00001). Thus, geographical distance is a relevant factor in the genetic differentiation of *colocola* populations. The correlograms showed a strong tendency towards a monotonic cline. Like the previous study, Ruiz-García et al. [145] considered the existence of a single species of Pampas cat. Two results were highly relevant in this regard. The levels of genetic heterogeneity for mtDNA in the different Pampas cat populations considered were very similar to those found, in parallel, in the Andean cat (*Leopardus jacobita*), a species considered monotypic, even with a smaller distribution range than the Pampas cat. Even for the Pampas cat, the levels of genetic heterogeneity in microsatellites, although significant, were considerably lower than those found among different populations of *L. jacobita*. This could mean that gene flow is biased towards males and that it goes undetected in studies carried out exclusively with mtDNA. A second finding obtained with microsatellites was the low rate of correct population assignment of Pampas cat specimens, as previously mentioned, when using the GENCLASS program (around 50% of the specimens were correctly assigned to their respective populations of origin, and there was a high proportion of first-generation migrants, unlike what occurred with *L. jacobita* where population assignment was much more robust). This highlights a high level of gene flow among *L. colocola* populations, with males acting as vectors of this flow. These results could indicate that we are dealing with a single species, at least regarding *L. pajeros* sensu García-Perea [142]. **(4)-** Santos et al. [147] analyzed 40 Pampas cat specimens, primarily from Brazil (and including a few individuals from Argentina, Chile, and Bolivia), for four mitochondrial markers. Additionally, they included some samples of tigrinas from Brazil (*L. tigrinus*). A Bayesian tree showed the existence of six well-defined groupings: (1) group 1 consisting of *L. colocola* specimens from central and northeastern Brazil, plus one tigrina specimen recently introgressed by the Pampas cat of this group; (2) group 2 of tigrinas from northeastern Brazil historically introgressed by the Pampas cats of group 1 about 144,000 years ago; (3) group 3 made up of Pampas cats from southern Brazil and Uruguay; (4) group 4 made up of tigrinas from northeastern and central Brazil historically introgressed about 110,000 years ago by the Pampas cat of group 3; (5) a group 5 consisting of three Chilean and one Bolivian specimens of Pampas cat, and (6) a group 6 consisting of two Chilean and two Argentinian specimens of Pampas cat. From this, it can be deduced that there was a colonization process of the Pampas cat from the west (Andean region) to the east (northern, central, and southern Brazil), and at least two population expansion events were recorded. One occurred approximately 200,000 years ago in western South America, and another more recent one in the Brazilian region (approximately 60,000 years ago), coinciding with the expansion of savanna environments in what is now Brazil. During this eastward expansion, the Pampas cat introduced its mtDNA to different populations of *L. tigrinus*. These authors continued to accept the existence of a single species of Pampas cat as had been done in previous molecular studies [127,144,145]. **(5)**- Nascimento et al., [140], using different criteria (morphological, molecular, biogeographical, and climatic niche data) concluded that there are five monotypic species of Pampas cats: *Leopardus braccatus* (eastern Bolivia, Paraguay and central and northeastern Brazil), *Leopardus colocola* (central Chile), *Leopardus garleppi* (including *thomasi*, *budini*, *steinbachi*, *crespoi* and *wolffsohni* as synonyms; Ecuador, Peru, Bolivia, northern Chile and northern Argentina), *Leopardus munoai* (southern Brazil and Uruguay; note in this case that Martínez-Lafranco and González [148] postulated that the correct name for this taxon would be *Leopardus fasciatus*, [149]) and *Leopardus pajeros* (including *cruscinus* as a synonym; central and southern Argentina). However, the molecular data they used do not seem entirely robust enough to determine the existence of these five supposed Pampas cat species because they have not demonstrated the existence of either post-zygotic or pre-zygotic reproductive isolation barriers between the different forms of this cat (biological species concept). They used a database with small and heterogeneous sample sizes for four mitochondrial genes. For the mt*ATP8* gene, they used seven individuals; for the control region, 38 individuals; for the mt*Cyt-b* gene, 12 individuals; and for the mt*ND5* gene, 24 individuals. With such a small and unequal sample, the authors found five distinct groups that they make correspond to the five species mentioned. However, one specimen of *L. colocola* was grouped with *L. pajeros*, one specimen of *L. garleppi* was grouped with *L. pajeros*, and one specimen of *L. pajeros* was grouped with *L. braccatus*. Previous analyses [144,145], using many more samples (albeit representative of only three of these taxa: *L. pajeros*, *L. garleppi*, and *L. colocola*), have shown that there are several areas where hybridization points are found among them, as well as with intermediate morphotypes. Furthermore, the divergence times estimated by Nascimento et al. [140] among these supposed five species of Pampas cat would have occurred approximately 540,000 years ago, a time that does not appear to be extremely long. On the other hand, Ruiz-García et al. [135] and Ruiz-García et al. (unpublished) have shown that, using individual mitochondrial genes and whole mitogenomes of Pampas cats from their entire distribution range, with the exception of Ecuador and possibly Colombia, they all form a compact and monophyletic clade when compared to the other species of the genus *Leopardus*. Similarly, the genetic distances between all Pampas cat taxa are less than the genetic distances between any other pair of full species of the genus *Leopardus*. Likewise, gene flow levels among most Pampas cat taxa using microsatellites showed substantially high gene flow (Nm) values, much higher than for any other pair of species of the genus *Leopardus*. Ruiz-García et al. [145] showed that gene flow in the Pampas cat is particularly mediated by males. Therefore, much more research is needed on the Pampas cat to confirm the existence of five fully differentiated species (whole genome sequencing is crucial).

Traditionally, *L. colocola thomasi* or *L. garleppi* had not been reported in Colombia. However, Ruiz-García et al. [150] detected an unusual feline pelt from southern Colombia (Nariño department), and although the molecular results were ambiguous, a likely explanation was that it belonged to the northernmost Pampas cat taxon, as this taxon extends into northern Ecuador [142]. Although this pelt ultimately did not belong to a Pampas cat, it was the first study to suggest the possible presence of this cat species in southern Colombia. During 2017–2018, a photograph began circulating (provided to the author of this text by Dr. González-Maya) that clearly showed a Pampas cat, phenotypically identified as having been run over on a Colombian road between the departments of Nariño and Cauca. This photograph demonstrated that, albeit occasionally, the Pampas cat does reach Colombian territory. It remains to be seen whether these incursions of animals from northern Ecuador into southern Colombia are occasional or if a permanent population of Pampas cats exists in southern Colombia. Astorquiza et al. [133] appear to have molecularly confirmed (using small segments of mtDNA) that the animal seen in the aforementioned photograph, which had been circulating in Colombia for years, is indeed a Pampas cat (the morphotype in the photograph is undeniably that of a Pampas cat). However, a population genetic study of the Pampas cat has never been conducted in Colombian or Ecuadorian territory. In fact, given the uncertainty surrounding whether there is truly only one species of Pampas cat (*L. colocola thomasi* or *L. colocola garleppi*, if it could be genetically demonstrated that these two taxa are synonymous) or whether the five species proposed by Nascimento et al. [140] (*L. garleppi*) exist, we still cannot determine the correct nomenclature to use for the Pampas cat in Colombia. It should be noted that, although Kitchener et al. [106] and Nascimento et al. [140] argue that *thomasi* is a synonym of *garleppi*, García-Perea [142] showed that *thomasi* is conspicuously smaller than *garleppi* and that its ectotympanic chamber is also smaller than that of *garleppi*. In fact, at present, the Pampas cat, if it is a permanent species in Colombian territory, could potentially be referred to by four different scientific names: *L. colocola thomasi*, *L. colocola garleppi*, *L. thomasi*, or *L. garleppi*. Therefore, an in-depth molecular genetic study of the Pampas cat in Ecuador and, eventually, in southern Colombia is essential to determine whether these taxa are synonymous and the implications this would have for the systematics of the Pampas cat. Table 7 shows a comparative analysis of some genetic and population parameters for the Pampas cat.

## 3. Conclusions

Therefore, the following conclusions can be drawn regarding the seven feline species that inhabit some portion of Colombian territory:

(1) A significant number of publications contain accurate and abundant genetic results for jaguars of Colombian origin, resulting in a good genetic understanding of the jaguar in this country. Potentially, molecular data exist for conducting studies at a micro-geographic level within Colombia. Furthermore, the levels of genetic diversity of the jaguar in Colombia appear to be among the highest reported compared to those found in other areas of this species’ distribution where its genetic variability is clearly lower (e.g., the Brazilian Atlantic Forest, Central America, and Mexico).

All mitochondrial studies have revealed very high genetic diversity in jaguars [16,56]. However, there is a clear predominance of studies using nuclear microsatellite loci in this species. As already mentioned, Amazonian jaguar populations exhibit the highest genetic variability [30,41,50,52,53] with these markers, while Mexican and Central American populations show more moderate levels of genetic diversity [27,28,29,36,39], probably due to their peripatric situation. Jaguars from the Brazilian Atlantic Forest show the greatest decline in their genetic diversity, primarily due to anthropogenic processes [23,38].

The Amazonian population is basically comprising a single population (Colombia, Ecuador, Peru, Bolivia, and Brazil), with perhaps also a single population in the Pantanal [18,24,25,30], and greater fragmentation into genetically distinct populations in Mexico and Central America (between one and four different populations) [27,28,29,36,39], and strong genetic heterogeneity among small remaining jaguar populations in the Brazilian Atlantic Forest [23,38].

At the global level, no significant spatial patterns appear to exist for the Amazon basin and the entire area of northern and central South America [41,50,52,53]. However, a significant spatial autocorrelation has been detected globally in this species [Ruiz-García et al., [56] and Ruiz-García et al. (unpublished results)] when comparing the area of northern and central South America with areas in the south of the continent (southern Bolivia and Paraguay) and with Central American populations from Guatemala [Ruiz-García et al., [56] and Ruiz-García et al. (unpublished results)]. Similarly, isolation by distance was found among jaguars in Mexico [36].

N_e_ estimates for jaguars have generally been obtained in very localized areas [23,38] and have always been small. Conversely, studies that have primarily included Amazonian specimens have revealed high N_e_ values [30,41,46]. Globally, for a single country, N_e_ estimates have only been found for the jaguar population of Colombia [50].

Globally, and for the Amazon, significant population expansions have been detected during various periods of the Pleistocene [16,52,53,56]. However, in other, smaller geographic areas, evidence of population bottlenecks has been detected. This is the case in Mexico [29,30], the Brazilian Caatinga region [30], and there are small indications of this in the Colombian trans-Andean jaguar population [50]. A population decline of jaguars in northwestern South America, beginning approximately 30,000 years ago, has also been detected [Ruiz-García, unpublished results].

Molecular studies do not clearly show the existence of jaguar subspecies. The most distinct populations are likely those in northern Central America and Mexico, and those in southern South America. It remains to be investigated whether these can be considered distinct subspecies, although the genetic differences are small.

Although two subspecies are recognized in Colombia (*P. onca centralis*, listed as Vulnerable (Vu), and *P. onca onca*, listed as Near Threatened (NT)), molecular studies do not differentiate between these two subspecies. However, the population of the former has shown signs of a population bottleneck, while the latter clearly exhibits symptoms of population expansion. At present, there are very complete genetic results (nuclear and mitochondrial markers) from 22 different departments of Colombia (Antioquia, Amazonas, Arauca, Atlántico, Bolívar, Boyacá, Caquetá, Casanare, Cauca, Chocó, Córdoba, Cundinamarca, Guainía, Guaviare, Huila, Magdalena, Meta, Norte de Santander, Putumayo, Valle del Cauca, Vaupés, Vichada; Colombia has 31 mainland departments, but some of them do not have jaguar populations); therefore, the genetic analyses of the jaguar in Colombia are very well represented.

(2) Conversely, although a significant number of genetic publications exist for the puma in the Neotropics (in addition to the abundant publications for this species in North America), only two of them have used Colombian specimens for comparison, and there are no publications dedicated exclusively to the study of the genetic structure of the puma in Colombia. Therefore, a study dedicated exclusively to determining the genetic and population parameters of the puma in Colombia is urgently needed.

In the case of the puma, the number of studies using mitochondrial markers (5 studies) and nuclear microsatellite loci (10 studies) is more balanced than in the case of the jaguar, where the latter are more prevalent. All studies (regardless of whether mitochondrial or nuclear markers were used) showed higher levels of genetic diversity in South American puma populations (with the exception of Patagonian pumas, which appear to have originated from a clear founder effect from populations further north in that country) [75], while Central American populations showed more moderate levels of genetic diversity, and North American puma populations clearly showed the lowest levels of genetic diversity [63,69,70]. This indicates that the origin of the modern puma is in South America and that its colonization of North America occurred relatively recently. The study by Culver et al. [63] identified a distinct North American group, a distinct Central American group, and four well-differentiated groups in South America. More recently, Matte et al. (70) identified seven well-defined groups (northern Central America + North America, southern Central America, northern South America, north-central South America, eastern South America, south-central South America, and southwestern South America). Therefore, the puma is distributed among a greater number of genetically distinct populations throughout its range compared to the jaguar, which is relevant to its biological conservation.

Globally, the spatial structure of the puma is significant [56,70], but within South America alone, there is no evidence of isolation by distance or spatial autocorrelation. In Mexico, isolation by distance was detected in its puma populations [36].

A single global estimate of N_e_ has been carried out for the puma [46], and a few have been done for some puma populations in the Brazilian Atlantic Forest and southern Brazil, which is the micro-geographic area that has been most intensively studied genetically for pumas in Latin America [65,66,67,68].

A significant proportion of genetic studies with pumas have revealed significant population expansions during the Pleistocene [56,70,75]. However, bottlenecks have been detected in more localized areas, such as certain parts of Colombia and southern Brazil.

Unlike the jaguar, where there are essentially no genetic results consistent with the putative morphological subspecies, in the case of the puma, six or seven genetically well-defined subspecies could be molecularly identified out of the 30 subspecies that have been morphologically defined. This is relevant for conservation purposes.

In Colombia, the puma is listed as near threatened (NT), but unlike the jaguar, there are no recent studies that thoroughly analyze its genetic structure in this country. The presence of the puma has been reported in all 31 mainland departments of Colombia. However, only partial genetic data are available for specimens from 11 Colombian departments (Amazonas, Atlántico, Bolívar, Boyacá, Guainía, Meta, Risaralda, Santander, Tuparro, Valle del Cauca, and Vaupés). Therefore, it is a priority to conduct a population genetic study of the puma with abundant samples representative of most of the country.

There are fewer publications with genetic analyses of the jaguarundi, ocelot, and margay compared to the molecular genetic research conducted on the jaguar and puma, especially for the first and third species. However, a significant portion of the specimens of these three species analyzed are of Colombian origin, thus providing important knowledge of their genetics within Colombian territory. All three species exhibited high levels of genetic diversity in Colombia and, in some cases (e.g., the ocelot), higher than those found in other areas of the Neotropics (Central America and Mexico) as is the case with the jaguar.

(3) Unlike the jaguar and puma, more studies using mitochondrial than nuclear markers exist for the jaguarundi, indicating an urgent need for further research using nuclear markers for this species. While several estimates of mitochondrial genetic diversity exist (and are very high in all studies), comparable estimates of nuclear genetic diversity are lacking (only one exists for various areas of South America) [44]. The jaguarundi population in northern Mexico is genetically impoverished compared to the South American population, due to its peripatric nature and negative anthropogenic influences.

Only two studies [56,93] have attempted to determine how many genetically distinct jaguarundi groups exist across most of their geographic range. Between four and six genetically heterogeneous populations have been identified. However, no evidence of isolation by distance or spatial autocorrelation, as observed in other Neotropical feline species, has been found for the jaguarundi. The genetic similarity between animals from the Colombian Caribbean coast and animals from the Argentine and Paraguayan Chaco is striking. There is no clear explanation for this similarity.

Only one study has attempted to estimate the N_e_ value on a macro-geographic scale. Unlike jaguars and pumas, for example, no micro-geographic N_e_ estimates exist for this species.

All demographic analyses carried out [46,56,91,93] have detected exclusively significant population expansions, except for some population decline over the last 20,000 years [91].

Most of the four to six genetically distinct populations do not correlate with the morphologically proposed subspecies.

In Colombia, this species is categorized as Least Concern (LC). The molecular results for this species are abundant and show the existence of high levels of genetic diversity (at least at the mitochondrial level) and the absence of a consolidated spatial structure, and thus these results seem to coincide with its categorization for this country. In Colombia, the jaguarundi is distributed throughout most of the country. Complete mitogenomes exist for specimens from 15 different departments (Amazonas, Arauca, Bolívar, Cauca, Cesar, Chocó, Córdoba, Guainía, Guajira, Huila, Meta, Risaralda, Sucre, Tolima, Vichada), ensuring significant genetic knowledge of this species in Colombia.

(4) In the case of the ocelot, studies estimating genetic diversity using microsatellites also predominate over those using mitochondrial markers, although this is not as pronounced as in the case of the jaguar. All studies conducted in South America (and Costa Rica) reveal high (or very high) levels of genetic diversity for both types of molecular markers. However, ocelots in northern Central America exhibit more moderate genetic diversity. In the case of populations in Texas (USA), genetic diversity levels are extremely low, primarily due to anthropogenic factors [111,112], although peripatric populations generally have lower levels of genetic diversity. Ocelots show some interesting genetic structuring across their entire geographic range. Microsatellite studies show consistent differentiation between South American and Central-North American populations, and within the latter, several distinct populations are detected [28,111]. Studies using mitochondrial markers have detected the existence of multiple local populations that exhibit slight differentiation among them. For example, in Colombia, at least seven genetically distinct populations have been identified [56]. This genetic structuring in the ocelot is noteworthy, as it is the largest species in the genus *Leopardus* and it should be the most mobile species of this genus. However, this species possesses much greater genetic heterogeneity among its populations than, for example, the margay, a smaller species much more specialized for arboreal life and, one might think, it has a lower dispersal capacity. This genetic differentiation among various ocelot populations should be relevant to conservation programs for this species.

Consistent with this, many studies have detected significant spatial autocorrelation and isolation by distance in the ocelot [28,56,115]. Only two studies [46,52] have estimated the effective global population size of the ocelot. Globally, these estimates appear high.

Microsatellite studies have detected population bottlenecks in various geographic areas (certain areas of Colombia, certain populations in the Colombian Amazon, southern Brazil, and northern Mexico and Texas) [46,52,111,112,114]. However, mitochondrial markers have revealed a strong population expansion globally during the Late Pleistocene.

Some of the 10 morphologically defined ocelot subspecies coincide with some of these local populations that exhibited some mitochondrial genetic differentiation (for example, *L. p. melanurus* with group 15 detected in [56], *L. p. pseudopardalis* with groups 12 and 13 detected in [56], *L. p. steinbachi* with group 7 detected in [56]), but the genetic differences are too small to consider them distinct subspecies. In general, the number of ocelot subspecies at the molecular level could be reduced to three or four.

In Colombia, the existence of two subspecies, *L. p. aequatorialis* and *L. p. pseudopardalis*, has traditionally been recognized, both classified as Near Threatened (NT). However, genetic results show that there are more distinct local populations that are not included in any conservation program and, in all cases, exhibit high levels of genetic diversity, although a slight bottleneck was detected in some of these groups, particularly those analyzed with microsatellites. In Colombia, the ocelot is potentially distributed across all 31 mainland departments. Extensive molecular results have been obtained for specimens from 23 different departments (Amazonas, Antioquia, Bolívar, Caldas, Caquetá, Casanare, Cauca, Cesar, Chocó, Córdoba, Cundinamarca, Guainía, Guaviare, Huila, Magdalena, Meta, Putumayo, Risaralda, Santander, Tolima, Valle del Cauca, Vaupés, Vichada), ensuring significant genetic knowledge of this species in Colombia.

(5) The margay exhibited very high levels of genetic diversity for both nuclear and mitochondrial markers. The few genetic studies conducted on this species are well distributed between nuclear and mitochondrial markers, although the most extensive studies (both in terms of the number of specimens analyzed and geographic distribution range) [56,117] used mitochondrial DNA, unlike what has been observed in jaguars or pumas, for example.

The genetic heterogeneity found among margay populations is less than that found for ocelots. Several studies found eight to 13 or 14 different groupings globally [56,117]. However, these groupings were of a different nature than those of ocelots. In the margay, most of these groupings were made up of specimens with different geographic origins and, therefore, are mixed groupings. In contrast, in the case of ocelots, the groups found are made up of specimens from specific and restricted geographic areas. Consistent with this finding, no spatial autocorrelation or isolation by distance was detected for the margay. Only one study [46] estimated N_e_ for the margay globally, obtaining a considerably high estimate, which overlaps with that of the ocelot. No study has detected any population bottlenecks in this species, but strong population expansions have been observed in the late Pleistocene [56,117].

Although some margay groups are genetically slightly different from each other, in general, there is no correspondence with any of the 10 morphological subspecies proposed for the margay.

In Colombia, three subspecies of margay have traditionally been recognized, *L. w. amazonicus*, *L. w. pirrensis*, and *L. w. vigens* and all three have been classified as Near Threatened (NT). However, all estimates of genetic diversity obtained for this species are very high, and to date, no evidence of bottlenecks has been detected in any Colombian margay population. In Colombia, the margay has been recorded in 26 departments. Abundant genetic results are available from specimens sampled in 11 departments of the country (Amazonas, Antioquia, Caquetá, Chocó, Guainía, Magdalena, Meta, Nariño, Norte de Santander, Valle del Cauca, Vichada), thus ensuring the genetic knowledge of this species in Colombia.

(6) In the case of tigrinas, the situation is complex. The only tigrina taxon that is well-analyzed at the genetic level is the southern Brazilian tigrina (*L. guttulus*) [128,129,130]. It showed high levels of genetic diversity for both nuclear and mitochondrial markers. For Andean tigrinas, two major groups have been identified [135] (G1 and G2). For these, genetic diversity estimates exist only for mitochondrial markers. These estimates were very high. However, within G1, there are likely two different taxa, and within G2, there may be several different taxa, thus these genetic diversity estimates may be inflated.

Within *L. guttulus*, there has clearly been genetic introgression and hybridization by *L. geoffroyi*, and in the tigrinas of central and northeastern Brazil, genetic introgression from *L. colocola* has also occurred. Genetic introgression from ocelots and margays has also been detected in the Andean tigrinas. Genetic introgression from *L. geoffroyi* has also been detected in Bolivian tigrinas. This phenomenon makes it difficult to determine how many species or taxa of tigrinas exist, which, in turn, hinders the development of effective conservation programs for tigrinas. Additionally, the discovery of specimens such as the Nariño cat and other distinct specimens found in Colombia and Ecuador that do not fit into either of the two major groups mentioned (G1 and G2) further complicates the understanding of the actual number of tigrina taxa in the Andes. Population expansion has been detected for *L. guttulus*, and a clear patchy spatial structure exists for Andean tigrina in Colombia and Ecuador. However, no estimates of N_e_ have been made for tigrinas, and the general lack of knowledge regarding their population genetics and systematics, except for *L. guttulus*, hinders conservation programs.

In Colombia, the only tigrina taxon traditionally recognized is *L. t. pardinoides*, and it has been classified as vulnerable (VU). However, this classification has little relevance because, in reality, we do not know how many tigrina taxa exist in the Northern Andes nor do we know the nomenclature that should be used. In Colombia, the Andean tigrina has been recorded in 21 departments. Mitochondrial DNA results are available from a relatively small number of specimens sampled in 12 departments (Antioquia, Boyacá, Caldas, Caquetá, Chocó, Cundinamarca, Huila, Meta, Nariño, Quindío, Risaralda, and Valle del Cauca), making it a species in Colombia where obtaining samples is not easy. Some progress has been made in understanding the genetics of the Andean tigrina, but further genetic studies are still needed.

(7) The population genetics of the Pampas cat are well known in countries such as Peru, Bolivia, Chile, Argentina, Brazil, and Uruguay. An exception in this region is the genetic study of this species in Paraguay. However, no genetic data exist for the Pampas cat in the northernmost part of Peru, Ecuador, or potentially Colombia, if a permanent population should exist there. In countries where there is a good genetic understanding of this species, levels of genetic diversity are high for both nuclear and mitochondrial markers.

Significant genetic heterogeneity exists among different populations of *L. colocola*. Some authors [140] consider this evidence for the existence of five different species of Pampas cat. However, other studies conclude that, despite this significant genetic heterogeneity, each of these populations should be considered a geographic subspecies of a single species (*L. colocola*), among other reasons, because all pairs of comparisons between these Pampas cat taxa with genetic distances are much lower than any pair of comparisons with all species included in the genus *Leopardus*. Furthermore, there are geographic areas where several of these taxa converge (southern Bolivia and northern Argentina, for example), and there is high gene flow mediated by males and many cases of intermediate morphological patterns [127,135,144,145,147]. Two studies have shown the existence of significant spatial autocorrelation for this species [56,145], and most of the reported studies indicate the existence of significant population expansions during the late Pleistocene [144,145,147]. Only for the *steinbachi* taxon in Bolivia was any sign of a bottleneck detected [145]. The genetic differences found are quite consistent with most morphologically defined taxa, at least at the subspecies level. Therefore, there is a greater correlation in this species, compared to the other species discussed, between morphologically defined taxa and molecular results.

For the Pampas cat, as mentioned above, we do not know whether there is a permanently established population in Colombia or if they are only individuals that occasionally cross the Colombian Ecuadorian border. Likewise, no population genetic study has ever been conducted on the Pampas cat in Ecuador, and therefore, we do not know whether *thomasi* and *garleppi* are synonymous. Furthermore, since we do not know exactly whether there is only one species of Pampas cat, or up to five different species, we cannot be certain of the correct scientific nomenclature to use for the Pampas cat found in Ecuador and, consequently, in Colombia. There are no genetic results for this species in Colombia or Ecuador, which greatly complicates any conservation program for this species in these countries.

Molecular analyses that can be carried out in the coming years will be fundamental to resolving these unknowns about the magnificent felines that inhabit Colombian fauna.

## Figures and Tables

**Table 1:** Comparative results of the most important population genetic studies carried out with the jaguar (*Panthera onca*). n = sample sizes; GA = Geographical area; mt = Mitochondrial; GH and NP = Genetic heterogeneity and number of populations; SS = Spatial Structure; PDCH = Possible Demographic Changes; N_e_ = Effective numbers; micro = microsatellite loci; H_d_ = Haplotype diversity; π = nucleotide diversity; H_e_ = Expected heterozygosity; n_A_ = average number of alleles per locus; PIC = polymorphism information content; ya = years ago. When possible, the standard deviations for these statistics were estimated.

Study	n	GA	mt Diversity	Nuclear Diversity	GH and NP	SS	PDCH	N_e_
Eizirik et al. [16]	37 (mt: 1 gen) 42 (micro: 29)	Global scale	H_d_ = 0.939 ± 0.026; π = 0.0077 ± 0.0001; High	H_e_ = 0.739; n_A_ = 8.31; high	mt: two weak separated populations by Amazon River; micro: two weak separated populations by Darién Gap	-	Expansion around 300,000 ya	-
Moreno et al. [17]	39 (micro: 4)	Brazilian zoos	-	PIC = 0.59 ± 0.125; n_A_ = 9 ± 3.67; high	-	-	-	-
Eizirik et al. [18]	23 (micro: 12)	Pantanal (Brazil)	-	H_e_ = 0.717; n_A_ = 5.83; moderately high	One population	-	-	-
Haag et al. [23]	50 (micro: 13)	Atlantic Forest (Brazil)	-	-	Different populations	-	-	Micro-scale: 4.6–51.4
Roques et al. [24]	16 and 14 (micro: 11)	Caatinga and Pantanal (Brazil)	-	H_e_ = 0.70 and H_e_ = 0.67; moderately high	Two different populations	-	-	-
Valdez et al. [25]	52 (micro: 12)	Pantanal (Brazil)	-	H_e_ = 0.70 ± 0.15; n_A_ = 6.55 ± 2.64 moderately high	One population	-	-	-
Wultsch et al. [27]	115 (micro: 12)	Mesoamerica (Mexico, Belize, Guatemala, Honduras, Costa Rica)	-	Overall: H_e_ = 0.59 ± 0.04; n_A_ = 4.50 ± 1.05; Extreme values: Mexico: H_e_ = 0.54; n_A_ = 3.25; Costa Rica: H_e_ = 0.64; n_A_ = 6.0; low and moderate	Four different populations	-	-	-
Wultsch et al. [28]	65 (micro: 14)	Belize	-	H_e_ = 0.57 ± 0.02; n_A_ = 3.80 ± 0.34; low and moderate	One population	-	-	-
Rueda-Zozaya et al. [29]	56 (micro: 11)	Mexican zoos	-	H_e_ = 0.65 ± 0.08; n_A_ = 5.03; moderate	Three different populations	-	Bottlenecks in global sample and in the tree different populations	13.4–22.7
Roques et al. [30]	78 (Brazil), 24 (Mexico and Belize) (micro: 11)	Amazon, Caatinga, Cerrado, and Pantanal (Brazil) and Mexico and Belize	-	Brazil: H_e_ = 0.812 ± 0.053; n_A_ = 9.45 ± 0.829; very high Mexico-Belize: H_e_ = 0.654 ± 0.147; n_A_ = 4.45 ± 0.325; moderate	Four different populations in Brazil; one population for Mexico and Belize	-	Bottlenecks in Caatinga (Brazil) and Mexico for IAM	Amazon:21–∞; Pantanal: 10–28; Caatinga: 7–28; Mexico: 14–45
Zanin et al. [36]	34 (micro: 11)	Mexico	-	H_e_ = 0.610 ± 0.036; n_A_ = 2.870 ± 0.279; moderate	Different procedures: undetermined number, eight or two populations	Isolation by distance	-	-
Srbek-Araujo et al. [38]	11 (micro: 11)	Espiritu Santo, Atlantic Forest (Brazil)	-	H_e_ = 0.532 ± 0.203; n_A_ = 3.45 ± 1.23; low	Different populations	-	-	7.9
Menchaca et al. [39]	50 (micro: 12)	Belize	-	H_e_ = 0.603 ± 0.207; n_A_ = 5 ± 2.16; moderate	One population	-	-	-
Lorenzana et al. [41]	73 (micro: 11)	Brazilian Amazon	-	H_e_ = 0.768 ± 0.134; n_A_ = 11.0 ± 5.67; very high	One population	No autocorrelation	-	241.4–∞
Ruiz-García [46]	24 (micro: 5)	Some specimens from northern Colombia and others from Colombian Amazon	-	H_e_ = 0.76 ± 0.29; n_A_ = 5 ± 1.4; high	-	-	No bottlenecks	Macro-scale: 105,000–307,000
Ruiz-García et al. [50]	62 (micro: 12)	Different areas of Colombia	-	H_e_ = 0.835 ± 0.075; n_A_ = 10.083 ± 2.571; very high	One population	-	No bottlenecks except for a possible recent bottleneck in the trans-Andean jaguar population	9755–24,084
Ruiz-García et al. [52]	107 (micro: 12)	Colombian Amazon; Peruvian Amazon; Bolivian Amazon	-	Like previous one; very high	One population	-	Population expansion	-
Ruiz-García et al. [53]	250 (mt: 3 genes) (micro: 12)	Global scale, although 156 jaguars of Colombian origin	-	Colombian Amazon: H_e_ = 0.867 ± 0.059, n_A_ = 13.0 ± 2.522; very high Peruvian Amazon: H_e_ = 0.883 ± 0.045; n_A_ = 8.083 ± 1.443; very high Bolivian Amazon: H_e_ = 0.883 ± 0.043; n_A_ = 8.75 ± 2.006; very high; Guatemala: H_e_ = 0.550 ± 0.034; n_A_ = 1.428 ± 0.787; low; Eastern Brazilian Amazon: H_e_ = 0.742 ± 0.123; n_A_ = 4.4 ± 1.776; moderately high	One population in Amazonas; different populations in other areas	-	Different population expansions in the Amazon	-
Jiménez-González et al. [55]	20 (micro: 9)	Colombian zoos	-	H_e_ = 0.684 ± 0.230; n_A_ = 5.67 ± 2.86; moderate	-	-	-	-
Ruiz-García et al. [56] and Ruiz-García et al. (unpublished)	157 (mt: 4 genes); 78 (whole mitogenomes); 112 (micro: 18)	Global scale, but Colombian, Ecuadorian, Peruvian, and Bolivian jaguars were highly represented	H_d_ = 0.995 ± 0.002, π = 0.0354 ± 0.003; very high (mt: 4 genes); H_d_ = 0.998 ± 0.0003, π = 0.0291 ± 0.0003; very high (mitogenomes)	H_e_ = 0.876 ± 0.041; n_A_ = 14.33 ± 2.91; very high	Eighteen and nine small clusters were detected in the first two databases, respectively, but these did not have much geographical significance. Geneland’s analysis detected seven genetically distinct populations for mitogenomes and four different populations for microsatellites, but these were mixed, in many cases, in overlapping geographic areas	Significant Isolation by distance at global scale, but not significant spatial pattern in northwestern South America	Mitochondrial DNA detected population expansions; microsatellites detected a sharp population decline in northwestern South America over the last 50,000–30,000 years. A Msvar analysis detected a significant population decline for jaguars in this region of South America over the last 3000 years.	-

**Table 2:** Comparative results of the most important population genetic studies carried out with the puma (*Puma concolor*). n = sample sizes; GA = Geographical area; mt = Mitochondrial; GH and NP = Genetic heterogeneity and number of populations; SS = Spatial Structure; PDCH = Possible Demographic Changes; N_e_ = Effective numbers; micro = microsatellite loci; H_d_ = Haplotype diversity; π = nucleotide diversity; H_e_ = Expected heterozygosity; n_A_ = average number of alleles per locus; PIC = polymorphism information content; ya = years ago. When possible, the standard deviations for these statistics were estimated.

Study	n	GA	mt Diversity	Nuclear Diversity	GH and NP	SS	PDCH	N_e_
Culver et al. [63]	315 (mt: 3 genes); (micro: 10)	Global scale	North America: π = 0.0002; low; Central America: π = 0.004; moderate; South America: π = 0.003; moderate	North America: H_e_ = 0.42 ± 0.016; low; Central America: 0.63 ± 0.011; moderate; South America: H_e_ = 0.71 ± 0.033; moderately high	North America: One population; Central America: One population; South America: Four populations	-	-	-
Ruiz-García [46]	50 (micro: 5)	Colombia (northern and Amazon); Peruvian Amazon; Santa Cruz department in Bolivia	-	H_e_ = 0.749 ± 0.243, n_A_ = 7.40 ± 2.10; high	-	-	Global and Colombian population showed some evidence of bottlenecks	Macro-scale: 74,600–185,900
Moreno et al. [17]	18 (micro: 4)	Brazilian zoos	-	PIC = 0.663 ± 0.167; n_A_ = 9.25 ± 2.86; moderately high	-	-	-	-
Ruiz-García et al. [64]	53 (micro: 7)	8 (Bolivian highlands); 45 (northwestern South America)	-	Bolivian sample: H_e_ = 0.942 ± 0.107; n_A_ = 3.86 ± 1.46; high; Northwestern South America: H_e_ = 0.845 ± 0.09; 11 ± 3.92; high	One population	-	-	-
Castilho et al. [65,66]	37 (micro: 18)	Southern Brazil	-	H_e_ = 0.682 ± 0.173; n_A_ = 5.888 ± 1.791; moderately high	One population	-	-	23.5
Miotto et al. [67]	111 (micro: 7)	São Paulo state (Brazil)	-	H_e_ = 0.797 ± 0.039; n_A_ = 9.286 ± 1.906; high	One population	-	Evidence of bottleneck	39.2
Saranholi et al. [68]	16 (micro: 7)	Tietê River in the Brazilian state of São Paulo	-	H_e_ = 0.77 ± 0.089; n_A_ = 7 ± 1.414; high	Two populations	-	-	-
Caragiulo et al. [69]	160 (mt: 4 genes)	Global scale	North America: two haplotypes and π = 0.0006; low; Central America: five haplotypes and π = 0.0017; moderate; South America: 11 haplotypes and π = 0.0022; moderately high	-	-	-	-	-
Matte et al. [70]	186 (mt: 1 gene)	Global scale, but especially South America	North America: H_d_ = 0.259 ± 0.156; π = 0.0022 ± 0.0016; low; Central America: H_d_ = 0.794 ± 0.075; π = 0.0052 ± 0.0011; moderate; South America: H_d_ = 0.904 ± 0.011; π = 0.0043 ± 0.0003; high	-	Seven populations	Globally, significant isolation by distance, but no spatial structure only for South America	Population expansions	
Wultsch et al. [28]	54 (micro: 14)	Belize	-	H_e_ = 0.57 ± 0.08; n_A_ = 4.46 ± 1.28; moderate	One population	-	-	-
Zanin et al. [36]	66 (micro: 12)	Mexico	-	H_e_ = 0.655 ± 0.074; n_A_ = 3.045 ± 0.850; moderate	Different procedures: two or five populations	Isolation by distance	-	-
Gallo et al. [73,74]	83 (micro: 25)	Central-southern Argentina	-	H_e_ = 0.713 ± 0.134; n_A_ = 6.9 ± 2.4; moderately high	Two populations	-	-	-
Ruiz-García et al. [56]	177 (mt: 4 genes)	Central and South America	H_d_ = 0.95 ± 0.013; π = 0.036 ± 0.003; high	-	Different populations	Isolation by distance	Population expansions and population decline in the last 15,000 ya	-
Mac Allister et al. [75]	180 (mt: 2 genes)	Central-southern Argentina	Central Argentina: H_d_ = 0.806 ± 0.079; π = 0.0048 ± 0.0013; high; Patagonia: H_d_ = 0.163 ± 0.054; π = 0.0003 ± 0.0006; very low	-	Two or three populations	-	Population expansions	-

**Table 3:** Comparative results of the most important population genetic studies carried out with the jaguarundi (*Puma = (Herpailurus) yagouaroundi*). n = sample sizes; GA = Geographical area; mt = Mitochondrial; GH and NP = Genetic heterogeneity and number of populations; SS = Spatial Structure; PDCH = Possible Demographic Changes; N_e_ = Effective numbers; micro = microsatellite loci; H_d_ = Haplotype diversity; π = nucleotide diversity; H_e_ = Expected heterozygosity; n_A_ = average number of alleles per locus; PIC = polymorphism information content; ya = years ago. When possible, the standard deviations for these statistics were estimated.

Study	n	GA	mt Diversity	Nuclear Diversity	GH and NP	SS	PDCH	N_e_
Ruiz-García [46]	16 (micro: 5)	Diverse South-American countries (Colombia, Venezuela, Peru, Bolivia and Brazil)	-	H_e_ = 0.616 ± 0.38; n_A_ = 4.6 ± 1.7; moderate	-	-	No population bottlenecks were detected	Macro-scale: 40,100–72,300
Moreno et al. [17]	36 (micro: 4)	Brazilian zoos	-	PIC = 0.825 ± 0.067; n_A_ = 10.75 ± 3.63; high	-	-	-	-
Ruiz-García and Pinedo [91]	44 (mt: 3 genes)	Diverse Latin American countries (Mexico, Guatemala, Costa Rica, Colombia, Venezuela, French Guiana, Ecuador, Peru, Bolivia, and Brazil	H_d_ = 0.960 ± 0.078; π = 0.055 ± 0.008; very high	-	No subdivision except for the Bolivian specimens	-	Population expansion (400,000 ya), but with a population decline in the last 20,000 years	-
Holbrook et al. [92]	11 (micro: 12) (mt: 1 gene)	Mexico	H_d_ = 0 ± 0; π = 0 ± 0; No mt diversity	H_e_ = 0.49 ± 0.22; n_A_ = 4 ± 1.65; low	-	-	-	-
Ruiz-García et al. [93]	80 (mt: whole mitogenomes)	Global scale	H_d_ = 0.995 ± 0.001; π = 0.0472 ± 0.0002; very high	-	With different procedures: six populations; five populations	No significant spatial autocorrelation	Population expansions (whole mitogenomes: 700,000 ya; control region: 300,000 ya)	-
Ruiz-García et al. [56]	80 (mt: 4 genes)	Global scale	H_d_ = 0.995 ± 0.004; π = 0.0473 ± 0.004; very high	-	Monmonier’s algorithm: four populations were detected	No significant spatial autocorrelation	Population expansion: 700,000–500,000 ya	-

**Table 4:** Comparative results of the most important population genetic studies carried out with the ocelot (*Leopardus pardalis*). n = sample sizes; GA = Geographical area; mt = Mitochondrial; GH and NP = Genetic heterogeneity and number of populations; SS = Spatial Structure; PDCH = Possible Demographic Changes; N_e_ = Effective numbers; micro = microsatellite loci; H_d_ = Haplotype diversity; π = nucleotide diversity; H_e_ = Expected heterozygosity; n_A_ = average number of alleles per locus; PIC = polymorphism information content; ya = years ago. When possible, the standard deviations for these statistics were estimated.

Study	n	GA	mt Diversity	Nuclear Diversity	GH and NP	SS	PDCH	N_e_
Eizirik et al. [100]	39 (mt: 1 gene)	Global scale	H_d_ = 0.962 ± 0.015; π = 0.068 ± 0.034; very high	-	four phylogeographic groups of ocelots	-	-	-
Ruiz-García [46]	68 (micro: 5)	Colombia and Peru	-	H_e_ = 0.837 ± 0.103; n_A_ = 10.00 ± 2.10; very high	One population	-	Bottleneck evidence in the Colombian and in the global sample	Macro-scale: 128,000–447,000
Grisolia et al. [101]	77 (micro: 4)	Brazilian zoos	-	H_e_ = 0.845 ± 0.036; n_A_ = 12.50 ± 0.87; very high	-	-	-	-
Ruiz-García et al. [52]	133 (micro: 12)	Costa Rica, Colombia, Venezuela, Brazil, French Guiana, Ecuador, Peru, Bolivia	-	H_e_ = 0.905 ± 0.124; n_A_ = 17.33 ± 4.21; very high	One population in the Colombian and Peruvian Amazon	-	Bottleneck in the Peruvian Amazon. Colombian Amazon population did not show evidence of population expansion	Macro-scale: 657,000–1, 176,000
Ruiz-García et al. [106]	294 (micro: 10)	Global scale	-	northwest South America: H_e_ = 0.906 ± 0.024; n_A_ = 15.8 ± 4.26; very high; Central America: H_e_ = 0.815 ± 0.184; n_A_ = 2.9 ± 1.37; moderately high	In South America, there is one unique population. Another possible population in Central America	-	-	-
Janecka et al. [111]	103 (micro: 25) (mt: 1 gene)	Two populations in Texas (USA) and one in Mexico	Cameron, Texas: H_d_ = 0, π = 0; no diversity. Tamaulipas (Mexico): H_d_ = 0.667; π = 0.0029; moderate	Cameron, Texas: H_e_ = 0.399; n_A_ = 2.88; low. Tamaulipas (Mexico): H_e_ = 0.637; n_A_ = 4.64; moderate	Three differentiated populations	-	-	-
Janecka et al. [112]	15 historical samples and 86 current samples (micro: 11) (mt: 1 gene)	Texas and Tamaulipas (Mexico)	Current diversity for Texas and Tamaulipas: H_d_ = 0.254 ± 0.060; π = 0.00077 ± 0.0002; low. Historical sample (1853–1956) for Texas and Tamaulipas: H_d_ = 0.543 ± 0.133; π = 0.00146 ± 0.00043; moderate	Current diversity for Texas: H_e_ = 0.389 ± 0.078; n_A_ = 2.46 ± 0.37; low. Historical Texas sample: H_e_ = 0.642 ± 0.034; n_A_ = 3.82 ± 0.33; moderate	-	-	-	-
Figueiredo et al. [114]	32 (micro: 9)	Southern Brazil		H_e_ = 0.709 ± 0.188; n_A_ = 5.777 ± 2.482; moderately high	One population	-	Evidence of bottleneck	-
Wultsch et al. [28]	30 (micro: 14)	Belize		H_e_ = 0.63 ± 0.03; n_A_ = 5.11 ± 0.15; moderate	One or two populations depending on the procedure	Significant, but weak isolation-by-distance		-
Salom-Pérez et al. [115]	28 (micro: 15)	Costa Rica	-	H_e_ = 0.79 ± 0.08; n_A_ = 6.87 ± 1.71; high	One population	Slight isolation by distance	-	-
Ruiz-García et al. [56] and Ruiz-García et al. (unpublished)	309 (mt: 4 genes) 340 (mt: 6 genes) 95 (whole mitogenomes)	Global scale	Four mt genes: H_d_ = 0.974 ± 0.005; π = 0.0306 ± 0.003; very high. Whole mitogenome: H_d_ = 0.993 ± 0.0004; π = 0.018 ± 0.00016; very high	-	Four genes: detected 10 populations; Six genes detected 15 different populations	significant isolation-by-distance structure over a range of 5000 km	Population expansion in the last 100,000–200,000 ya	-

**Table 5:** Comparative results of the most important population genetic studies carried out with the margay (*Leopardus wiedii*). n = sample sizes; GA = Geographical area; mt = Mitochondrial; GH and NP = Genetic heterogeneity and number of populations; SS = Spatial Structure; PDCH = Possible Demographic Changes; N_e_ = Effective numbers; micro = microsatellite loci; H_d_ = Haplotype diversity; π = nucleotide diversity; H_e_ = Expected heterozygosity; n_A_ = average number of alleles per locus; PIC = polymorphism information content; ya = years ago. When possible, the standard deviations for these statistics were estimated.

Study	n	GA	mt Diversity	Nuclear Diversity	GH and NP	SS	PDCH	N_e_
Eizirik et al. [100]	24 (mt: 1 gene)	Global scale	H_d_ = 0.985 ± 0.018; π = 0.183 ± 0.092; very high	-	Three different populations (Central America; northeastern South America; south of the Amazon River)	-	-	-
Ruiz-García [46]	14 (micro: 5)	Colombia and Bolivia	-	H_e_ = 0.846 ± 0.140; n_A_ = 5.50 ± 1.54; very high	-	-	No evidence of bottlenecks	Macro-scale: 152,400–720,400
Grisolia et al. [101]	25 (micro: 4)	Brazilian zoos	-	H_e_ = 0.847 ± 0.048; n_A_ = 11 ± 1.58; very high	-	-	-	-
Pinedo and Ruiz-García [117]	118 (mt: 3 genes)	Global scale	H_d_ = 0.976 ± 0.009; π = 0.035 ± 0.0032; very high	-	Different procedures revealed 13 or 14 different populations, but many of them appeared intermingled in the same geographical areas	No spatial genetic structure was found	Population expansions in the last 400,000–200,000 ya	-
Ruiz-García et al. [56]	118 (mt: 4 genes)	Global scale	Same results as in the previous work	-	Same results as in the previous work	Same results as in the previous work	Same results as in the previous work	-

**Table 6:** Comparative results of the most important population genetic studies carried out with the tigrina (*Leopardus tigrinus* or similars). n = sample sizes; GA = Geographical area; mt = Mitochondrial; GH and NP = Genetic heterogeneity and number of populations; SS = Spatial Structure; PDCH = Possible Demographic Changes; N_e_ = Effective numbers; micro = microsatellite loci; H_d_ = Haplotype diversity; π = nucleotide diversity; H_e_ = Expected heterozygosity; n_A_ = average number of alleles per locus; PIC = polymorphism information content; ya = years ago. When possible, the standard deviations for these statistics were estimated.

Study	n	GA	mt Diversity	Nuclear Diversity	GH and NP	SS	PDCH	N_e_
Johnson et al. [127]	32 (mt: 3 genes)	Costa Rica and Brazil	-	-	More than 5% of genetic differences between Costa Rica and Brazilian tigrinas. Two possible species. Hybridization of Brazilian tigrinas with *L. colocola*	-	-	-
Trigo et al. [128]	57 (mt: 3 genes) (micro: 9)	Brazil	H_d_ = 0.927 ± 0.025; π = 0.0039 ± 0.00227; high	H_e_ = 0.716 ± 0.141; n_A_ = 8.89 ± 4.507; high	The southern Brazilian tigrine is a new species: *Leopardus guttulus*; Hybridization with *L. colocola* and *L. geoffroyi*	-	Population expansions	-
Ruiz-García et al. [131]	41 (mt: 2 genes); 18 (whole mitogenomes)	Costa Rica, Colombia, Venezuela, Ecuador, Peru, Bolivia	-	-	Four different populations or lineages: one introgressed with mtDNA of ocelots and margays, and a possible new taxon: *Leopardus narinensis*	-	-	-
Ruiz-García et al. [135]	44 (mt: 1 gene); 37 (whole mitogenomes)	Costa Rica, Colombia, Venezuela, Ecuador, Peru, Bolivia	Two main populations (G1 and G2); G1 for mt*ND5*: H_d_ = 0.958 ± 0.036; π = 0.0188 ± 0.0051; high; G2 for mt*ND5*: H_d_ = 1.000 ± 0.045; π = 0.0583 ± 0.0126; very high; G1 for whole mitogenomes: H_d_ = 0.949 ± 0.005; π = 0.0101 ± 0.00018; high; G2 for whole mitogenomes: H_d_ = 1.000 ± 0.005; π = 0.0594 ± 0.0006; very high	-	For the mt*ND5* gene, up to six different groupings (excluding the Nariño cat); For the whole mitogenomes, up to eight groupings (excluding the Nariño cat)	Significant spatial patterns	-	-

**Table 7:** Comparative results of the most important population genetic studies carried out with the Pampas cat (*Leopardus colocola*). n = sample sizes; GA = Geographical area; mt = Mitochondrial; GH and NP = Genetic heterogeneity and number of populations; SS = Spatial Structure; PDCH = Possible Demographic Changes; N_e_ = Effective numbers; micro = microsatellite loci; H_d_ = Haplotype diversity; π = nucleotide diversity; H_e_ = Expected heterozygosity; n_A_ = average number of alleles per locus; PIC = polymorphism information content; ya = years ago. When possible, the standard deviations for these statistics were estimated.

Study	n	GA	mt Diversity	Nuclear Diversity	GH and NP	SS	PDCH	N_e_
Johnson et al. [127]	22 (mt: 3 genes)	Brazil, Uruguay, Chile, Bolivia, Argentina	-	-	Three different populations (Argentina and central Chile; Bolivia and northern Chile; Uruguay and southern Brazil). Only 2.3% of genetic differentiation among the three populations. One species	-	-	-
Cossíos et al. [144]	199 (mt: 3 genes) (micro: 5)	Peru, Bolivia, Argentina	H_d_ = 0.94 ± 0.034; π = 0.0609 ± 0.0173; very high	H_e_ = 0.836 ± 0.043; n_A_ = 14.80 ± 3.544; very high	Four populations	-	Population expansion: 600,000–300,000 ya	-
Ruiz-García et al. [145]	235 (mt: 1 gene) (micro: 5)	Peru, Bolivia, Argentina, Chile	H_d_ = 0.932 ± 0.007; π = 0.0513 ± 0.0016; very high	H_e_ = 0.741 ± 0.064; n_A_ = 7.6 ± 3.48; very high	Significant genetic heterogeneity among five putative subspecies of *L. colocola*, but a unique species	Significant autocorrelation	Population expansions. For *steinbachi*, some evidence of bottlenecks	Macro-scale: 82,800–328,000
Santos et al. [147]	40 (mt: 4 genes)	Brazil, and some specimens from Argentina, Chile, and Bolivia	-	-	Four different populations. One species	-	Population expansions	-
Nascimento et al., [140]	7 (mt: *ATP8*); 38 (mt: control region); 12 (mt: *Cyt-b*); 24 (mt: *ND5*)	Like previous studies	-	-	Five different species	-	-	-

## Data Availability

No new data were created or analyzed in this study. Data sharing is not applicable to this article.
